# Biomarkers of environmental manganese exposure and associations with childhood neurodevelopment: a systematic review and meta-analysis

**DOI:** 10.1186/s12940-020-00659-x

**Published:** 2020-10-02

**Authors:** Weiwei Liu, Yongjuan Xin, Qianwen Li, Yanna Shang, Zhiguang Ping, Junxia Min, Catherine M. Cahill, Jack T. Rogers, Fudi Wang

**Affiliations:** 1 Department of Nutrition, Precision Nutrition Innovation Center, School of Public Health, Zhengzhou University, Zhengzhou, China; 2 The First Affiliated Hospital, School of Public Health, Institute of Translational Medicine, Zhejiang University School of Medicine, Hangzhou, China; 3 Neurochemistry Laboratory, Department of Psychiatry-Neuroscience, Massachusetts General Hospital and Harvard Medical School, Charlestown, MA USA

**Keywords:** Manganese exposure, Biomarker, Cognitive function, Behavior, Motor

## Abstract

**Background:**

Although prior studies showed a correlation between environmental manganese (Mn) exposure and neurodevelopmental disorders in children, the results have been inconclusive. There has yet been no consistent biomarker of environmental Mn exposure. Here, we summarized studies that investigated associations between manganese in biomarkers and childhood neurodevelopment and suggest a reliable biomarker.

**Methods:**

We searched PubMed and Web of Science for potentially relevant articles published until December 31th 2019 in English. We also conducted a meta-analysis to quantify the effects of manganese exposure on Intelligence Quotient (IQ) and the correlations of manganese in different indicators.

**Results:**

Of 1754 citations identified, 55 studies with 13,388 subjects were included. Evidence from cohort studies found that higher manganese exposure had a negative effect on neurodevelopment, mostly influencing cognitive and motor skills in children under 6 years of age, as indicated by various metrics. Results from cross-sectional studies revealed that elevated Mn in hair (H-Mn) and drinking water (W-Mn), but not blood (B-Mn) or teeth (T-Mn), were associated with poorer cognitive and behavioral performance in children aged 6–18 years old. Of these cross-sectional studies, most papers reported that the mean of H-Mn was more than 0.55 μg/g. The meta-analysis concerning H-Mn suggested that a 10-fold increase in hair manganese was associated with a decrease of 2.51 points (95% confidence interval (CI), − 4.58, − 0.45) in Full Scale IQ, while the meta-analysis of B-Mn and W-Mn generated no such significant effects. The pooled correlation analysis revealed that H-Mn showed a more consistent correlation with W-Mn than B-Mn. Results regarding sex differences of manganese associations were inconsistent, although the preliminary meta-analysis found that higher W-Mn was associated with better Performance IQ only in boys, at a relatively low water manganese concentrations (most below 50 μg/L).

**Conclusions:**

Higher manganese exposure is adversely associated with childhood neurodevelopment. Hair is the most reliable indicator of manganese exposure for children at 6–18 years of age. Analysis of the publications demonstrated sex differences in neurodevelopment upon manganese exposure, although a clear pattern has not yet been elucidated for this facet of our study.

## Background

Environmental metal exposure normally occurs in co-exposure to multiple metals, such as lead, cadmium, arsenic, mercury, chromium and manganese. Among these metals, manganese (Mn) is an essential trace element [1], but it is toxic, especially for brain functions, when abnormally deposition occurs in the body [2].

Growing interest has been recently generated to understand environmental manganese exposure in children [3, 4]. Meta-analysis about autism spectrum disorder (ASD) indicated that the mean difference in blood and hair manganese concentrations between ASD and control individuals was not significant [5]. In terms of neurocognitive development, these epidemiological studies had inconsistent conclusions across different biomarkers [6–9], which also left open the question as to whether there exists a useful biomarker for Mn exposure.

Evidence-based studies have also evaluated this association between manganese in hair and childhood IQ [10]. However, no comprehensive meta-analysis has been performed to examine Mn associations between different indicators and neurodevelopment. Thus, to the best of our knowledge, no meta-analysis has been performed regarding the putative correlation between such Mn indicators. Compared with cognition, the impacts of Mn on behavioral and motor development in children have been less evaluated, although Mn-related motor changes, such as in manganism, have been evaluated more extensively in occupational exposures [11, 12]. In addition, the potential for sex difference in the consequences of manganese exposure has also drawn attention, as there may be some differences between males and females in patterns of exposure, gastrointestinal absorption of chemicals, metabolism and detoxification [13].

To address these research gaps, the goal of this systematic review and meta-analysis has been to summarize and quantify the scientific evidence through different biomarkers or sources in order to obtain a clearer understanding of the exposure-response relationship between Mn indicators (biomarkers or environmental samples) and neurodevelopmental outcomes. In addition, we performed meta-analyses to seek a pooled correlation between Mn indicators (hair, blood and drinking water) and, here, suggested a potential biomarker for further epidemiologic studies of the toxic impact of Mn in childhood neurodevelopment. We also performed a preliminary meta-analysis to quantify the sex difference between manganese indicators and intelligence. Our conclusions provide useful suggestions for future public health studies, especially on the consequences of heavy metal exposures, such as Mn, towards human health.

## Methods

### Search strategy and inclusion criteria

Our study was conducted according to the Preferred Reporting Items for Systematic Reviews and Meta-Analysis (PRISMA) Statement. The completed PRISMA checklist is provided in Additional file 1. This systematic review protocol was registered with PROSPERO (CRD42020182284). Two investigators (authors W.L. and Y.X.) independently conducted a literature search in PubMed and Web of Science for studies published through December 31th 2019 in English, using the following search terms: (“manganese” or “manganism” or “manganese exposure”) and (“children” or “child” or “infant” or “childhood” or “adolescents” or “early life” or “young” or “younger populations”) and (“neurotoxicity” or “neuropsychological effects” or “neurodevelopmental outcomes” or “cognition” or “cognitive” or “intellectual function” or “intellectual impairment” or “intelligence quotient” or “IQ” or “memory” or “attention” or “mental” or “academic performance” or “hyperactivity” or “behavior” or “hyperactive behaviors” or “neurobehavior” or “motor” or “neuromotor”). In addition, the references included in relevant articles were searched for additional eligible publications.

Studies included in this systematic review had to meet the following criteria of being: (1) An original peer reviewed article; (2) A study of populations up to 18 years of age; (3) Manganese exposure was assessed through medicinal biomarkers (i.e. hair and blood) or environmental samples (i.e. drinking water); (4) A study of neurodevelopment derived from manganese exposure, including: cognitive, behavioral and/or motor changes; (5) Potential confounders were adjusted in the mathematical model for the estimated association between Mn indicator and a specific neurological outcome in children.

For inclusion in the meta-analysis, studies had to satisfy the above criteria and had to have measured the effect of manganese exposure on neurodevelopment by regression models, while for correlation analysis, the correlation coefficient (*r*) was provided. We excluded studies about attention deficit hyperactivity disorder (ADHD), which was reviewed in a recent paper, and the results of which showed higher peripheral manganese concentrations in children diagnosed with ADHD than those in controls [14]. We did not exclude articles published using the same population with different neurodevelopmental assessments [15, 16].

### Data extraction and quality assessment

The following information was extracted by two investigators (W.L. and Y.X.) independently using a standardized data collection form: first author, publication year, biomarker, country/study name, study design, sample size, age, sources of manganese exposure, neurological assessments and neurodevelopmental outcomes. For meta-analysis, the regression coefficient (*β*) with its 95% confidence interval (CI) and correlation coefficient (*r*) were also extracted. In the event of multiple articles published using the same population when assessing neurodevelopmental outcomes at different ages, and the same data were used in more than one publication, we consistently selected the most informative article, which was usually the most recent publication.

The guideline for Strengthening the Reporting of Observational Studies in Epidemiology (STROBE) was applied to assess the methodological quality of each study by two investigators (W.L. and Y.X.) independently [17]. The STROBE Statement is a checklist of 22 items that was initially developed to evaluate the systematic clarity in communicating research results in observational studies. This checklist has been used in systematic reviews to evaluate the methodological quality of observational studies [10, 18]. Nine items in methods (items 4–12) were selected, which covering the different aspects of methodology in observational studies. The methodological quality was classified by the number of items that the research met. To be more specific, articles that met 0–3 items, 4–6 items and 7–9 items were regarded as low, moderate and high methodological quality, respectively. Any disagreements were resolved by group discussion with a third investigator (Q.L.).

### Statistical analysis

A regression coefficient (*β*) with corresponding 95% CI was used as the common measure of association across studies [6–8, 16, 19–21]. A study that stratified by sex was treated as two separate reports [20]. We used a random-effects model to calculate the summarized *β* metrics and their corresponding 95% CIs. The meta-analysis was restricted to studies that used the Wechsler scales to evaluate IQ and linear regression models to examine the relationships between manganese exposure and children’s IQ scores. One study exhibited the scores of estimated IQ, vocabulary, block design and digit span, which were subtests from the Wechsler Intelligence Scale [22]. We took the scores of estimated IQ, block design and vocabulary as Full Scale IQ, Performance IQ and Verbal IQ, respectively [23].

Three manganese exposure metrics were included: hair, blood and drinking water. Furthermore, the *β* metric was estimated through different expressions of manganese concentration: *log*_*10*_, *log*_*2*_, *log*_*e*_ or non-transformation. We unified the expression as a *log*_*10*_-transformation to mean that the change in IQ (*β*) was associated with a 10-fold increase in the manganese exposure indicator, while we did not transform the *β* in blood, which was transformed using *log*_*e*_ consistently.

More specifically, in a linear regression model where the manganese concentration (*x*) was transformed by logarithm base 2 to correct the skewness of the data distributions, we expressed it into a *log*_*10*_-transformation by the formula *log*_*2*_^*x*^ * *β* = *log*_*10*_^*x*^ * *β1* to obtain a new coefficient (*β1*). The *β1* was approximately equal to 3.32 * *β*. Two studies assessed the effect of manganese exposure with raw manganese concentration. We used the similar formula to transform it into the changes in base *log*_*10*_. Clearly, the *β1* was equal to E(*x*)* *β*. E(*x*) was a function about the mean of manganese concentration (*x*), more specifically, E(*x*) = *x/log*_*10*_^*x*^*.*

In addition, a meta-analysis of correlation coefficients was also performed. Firstly, the Fisher’s *z* transformation was used to transform data as below,

1 $$ \mathrm{Fisher}\hbox{'}\mathrm{s}\kern0.5em z=0.5\ast \mathrm{In}\frac{1+r}{1-r} $$

2 $$ \mathrm{SE}=\sqrt{\frac{1}{n-3}}\;\left(n\;\mathrm{is}\ \mathrm{the}\ \mathrm{size}\ \mathrm{of}\ \mathrm{the}\ \mathrm{sample}\right) $$

3 $$ \mathrm{summary}\kern0.5em r=\frac{e^{2z}-1}{e^{2z}+1}\kern0.5em \left(z\;\mathrm{was}\ \mathrm{the}\ \mathrm{summary}\ \mathrm{Fisher}\hbox{'}\mathrm{s}\ z\right) $$

Then, we put the Fisher’s *z* and Standard Error (SE) into RevMan 5.3 using the generic inverse variance random effects model to obtain the summary Fisher’s *z*. Finally, the formula 3 was used to estimate the summary *r* [24]*.*

The meta-analysis was performed using Stata version 14 for regression and RevMan 5.3 for correlation. Heterogeneity among studies was estimated using the *I*^*2*^ statistic [25]. A “leave-one-out” sensitivity analysis and subgroup analysis based on the source of exposure were also performed. Publication bias was assessed using Egger’s test with a significant value set to *p* <  0.10 [26]. Except where noted otherwise, differences with a *p*-value < 0.05 were considered significant.

## Results

**Fig. 1:**
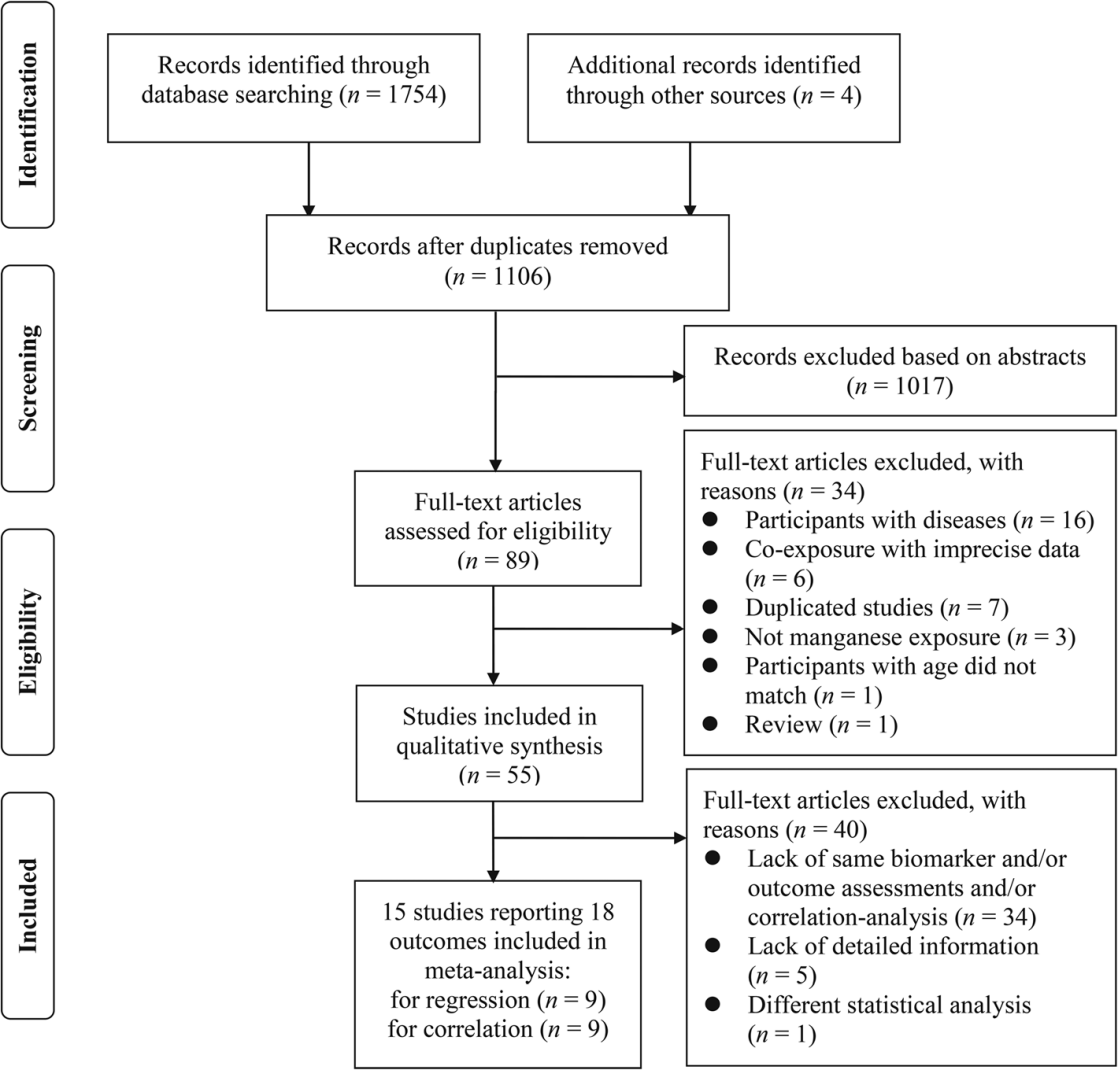
PRISMA flow diagram

A total of 1754 potentially relevant studies were identified through database searches (see Fig. 1). After applying the stringent inclusion and exclusion criteria described in the methods section, 55 original studies encompassing 13,388 children were ultimately included. Fifteen studies reporting 18 outcomes were included in the meta-analysis, with 9 studies for regression and 9 studies for correlation (see Fig. 1).

The sources of manganese exposure were mainly from industrial activities (i.e. metallurgy and mining) and drinking water (see Tables 1, 2, 3). More studies examined postpartum manganese exposure than prenatal exposure, meanwhile there were also some studies that measured manganese exposure from prenatal to postnatal periods. The concentrations of manganese were more frequently measured in biomarkers (*n* = 52, i.e. hair, blood and teeth) than environmental samples (*n* = 21, i.e. drinking water, particulate matter and soil). The associations between manganese in biomarkers and neurodevelopmental outcomes were investigated in 15 cohort studies and 37 cross-sectional studies (see Tables 1, 2). Table 3 presents the associations between manganese in drinking water (W-Mn) and neurodevelopment.

**Table 1:** Neurodevelopmental outcomes of manganese exposure mainly prenatal exposure measured in biomarkers from cohort studies

Author, Year	Age (Years)	Country/ Study Name	Number (Girls/Boys)	Sources	Biomarkers	Neurological Assessments	Associations between Manganese in Biomarkers and Neurodevelopmental Outcomes	Adjustment for Covariates	Study Quality
Chung 2015 [27]	0.5	Korea/MOCEH	232 (124/108)	NA	Maternal blood	BSID-II	An inverted U-shaped: mental and psychomotor development	Maternal age, gestational age, parity, income, breastfeeding status, maternal total calorie intake, residential area, infant sex and birth weight	High
Claus Henn 2010 [28]	1–2	Mexico	448^#^	NA	Blood	BSID-II	An inverted U-shaped: mental development at 1 year of age, 2 years of age: NS	Blood lead, sex, maternal IQ and education, hemoglobin and gestational age	High
Claus Henn 2017 [29]	2	USA	224 (91/133)	Mining	Maternal blood, cord blood	BSID-II	Maternal blood: ↓: mental and psychomotor development, cord blood: NS	Maternal age, smoking, gestational period, marital status, parity, income, and prenatal vitamin use	High
Freire 2018 [30]	4–5	Spain	302 (86/216)	NA	Placenta	MSCA	Placental Mn: ↓: perceptual-performance function, ↑: memory span and quantitative skills	Child’s sex, psychologist, child age, social class, maternal smoking during pregnancy and pre-pregnancy BMI	High
Gunier 2015 [31]	0.5, 1, 2	USA/CHAMACOS	197 (113/84)	NA	Teeth	BSID-II	Postnatal T-Mn: ↓: mental development at 6-months and at 12-months of age	Child’s age, sex, maternal education, IQ, psychometrician, location of assessment, household poverty and HOME score. Postnatal models also adjusted for prenatal Mn	High
Lin 2013 [32]	2	China/TBPS	230 (102/128)	NA	Cord blood	CDIIT	Cord blood: ↓: cognition and language	Maternal age, education, fish intake, sex, passive smoking and HOME score	High
Mora 2018 [33]	1	Costa Rica/ISA	355 (177/178)	Mancozeb	Maternal blood and hair	BSID-III	Maternal hair: ↓: cognition in girls, maternal blood: NS	Maternal education, parity, gestational period, child age, HOME score and location of assessment	High
Takser 2003 [34]	0.7, 3, 6	France	195, 126, 100 (44/56)	NA	*	MSCA	Cord blood: ↓: attention, non-verbal memory, hand skill at 3 years old, the other biomarkers: NS	Child’s sex and mother’s education	Medium
Yu 2014 [35]	Newborns	China	933 (439/494)	NA	Cord serum	NBNA	Cord serum Mn: ↓: fetal neurobehavioral development	Maternal age, education, occupation, incomes, birth weight, passive smoking, gestational age, sex, Pb and Hg	High
Yu 2016 [36]	1	China/LW birth cohort	377 (188/189)	NA	Cord serum	GDI	Cord serum Mn: ↓: gross motor and personal-social tasks	Maternal education, income, birth weight, Hg and Fe	High
Claus Henn 2018 [37]	6–16	Mexico/ELEMENT	138 (74/64)	Air pollution and diets	Teeth	WRAVMA	NS, stratified by sex, postnatal T-Mn: ↓: visual spatial scores in boys only	Child’s sex, tooth Pb levels, maternal IQ, maternal education and study cohort	High
Dion 2018 [20]	10.5–18	Canada	287 (151/136)	Ground water	Hair	WASI	Hair: NS, water Mn increased, Performance IQ scores decreased	Maternal IQ, education and income	High
Mora 2015 [38]	7, 9, 10.5	USA/CHAMACOS	248 (140/108)	Mancoze, maneb	Teeth	BASC-2 WISC-IV	Prenatal and postnatal T-Mn: ↓: behavior in boys and girls,↑: motor, memory and cognition in boys	Maternal education, IQ, years in the US, and depression at time of assessment, child’s sex and age, language of maternal interview, HOME score, income and number of children in the home at time of assessment	High
Wasserman 2016 [19]	12.4 ± 0.8	Bangladesh	296	Deep well water with reduced Mn	Blood	WISC-IV	Baseline B-Mn: ↓: working memory, reductions in B-Mn did not translate into improvements in child IQ	Maternal IQ and age, HOME score, child’s school grade, head circumference and plasma ferritin	High
Zhou 2019 [39]	6–8	China/SMBCS	296 (126/170)	NA	Cord blood, urine	WISC	Urinary Mn: ↑: Performance IQ in girls	Child sex, maternal age, education, income, inhabitation area and passive smoking	High

^#^: 1 year: *n* = 270 (131/139); 2 years: *n* = 430 (211/219); *:Maternal blood and hair, cord blood, newborns hair, placenta; ↓: Negative association; ↑: Positive association; *NA* Not available; *NS* No significant association. *Fe* Iron; *Hg* Mercury; *Mn* Manganese; *Pb* Lead. *B-Mn* Manganese in blood; *T-Mn* Manganese in teeth. *BMI* Body mass index; *HOME score* Home observation for measurement of the environment score; *IQ* Intelligence Quotient. *CHAMACOS* The Center for the Health Assessment of Mothers and Children of Salinas study; *ELEMENT* Early Life Exposures in MExico and NeuroToxicology; *ISA* Infantes y Salud Ambiental; *MOCEH* The Mothers and Children’s Environmental Health study; *SMBCS* Sheyang Mini Birth Cohort Study; *TBPS* The Taiwan Birth Panel Study

**Table 2:** Neurodevelopmental outcomes of manganese exposure measured in biomarkers from cross-sectional studies

Author, Year	Age (Years)	Country/ Study Name	Number (Girls/Boys)	Sources	Biomarkers	Neurological Assessments	Associations between Manganese in Biomarkers and Neurodevelopmental Outcomes	Adjustment for Covariates	Study Quality
Al-Saleh 2019 [40]	0.2–1	Saudi Arabia	206 (96/110)	NA	Maternal blood and urine, infant urine, breast milk	DDST-II, PEDS	NS	Maternal age and BMI, infant’s age, sex, parity, the location of primary health care centers, maternal education and *z* score weight for age	Medium
Rink 2014 [41]	1.1–3.7	Uruguay	60 (34/26)	NA	Hair	BSID-III	H-Mn: NS	HOME score, age, child Hb, maternal IQ, SES, Pb, marital status, father education and tester	High
Bauer 2017 [42]	10–14	Italy/PHIME	142 (79/63)	Fe-Mn alloy plant	Teeth	VRAM	Both low and high prenatal Mn ↓: visuospatial learning and working memory among girls only	Sex, age, SES, videogame use, lead, trial and tooth attrition	High
Betancourt 2015 [43]	11	Ecuador	93 (46/47)	Water consumption from the river	Hair	PCM	H-Mn: ↓: IQ	Mother’s literacy	Medium
Bhang 2013 [44]	8–11	Korea*	1001 (474/527)	NA	Blood	WASI, ADS, CBCL	B-Mn: ↓: academic performance, such as thinking, reading, calculation, lower Mn ↓: attention	Age, sex, region, children’s IQ, maternal education and age, levels of cotinine and lead	High
Bouchard 2007 [45]	6–15	Canada	46 (22/24)	Ground water	Hair	CPRS-R, CTRS-R	H-Mn: ↓: behaviors (teacher-reported hyperactive and oppositional behaviors)	Child’s age, sex and income	High
Bouchard 2011 [16]	6–13	Canada	362 (194/168)	Ground water	Hair	WASI	H-Mn: ↓: IQ	Maternal education and IQ, income, home stimulation score, family structure, sex and age of child and IQ testing session, source of water and level of iron in tap water	High
Bouchard 2018 [8]	6–14	Canada	259 (132/127)	Ground water	Hair, saliva, toe nail	WISC-IV	NS, possible beneficial effects in boys	Child’s age, maternal IQ and education, income and IQ tester	High
Carvalho 2014 [22]	7–12	Brazil	70 (36/34)	Air emissions from Fe-Mn alloy plant	Hair	WISC-III	H-Mn: ↓: estimated IQ, Block Design and Digit Span	Maternal education	High
Carvalho 2018 [46]	7–12	Brazil	70 (36/34)	Air emissions from Fe-Mn alloy plant	Hair	NEPSY II	H-Mn: ↓: verbal memory, behaviors (hyperactivity), not motor	Age, sex, SES, mother’s education and mother’s IQ	High
Chan 2015 [47]	11–13	USA/NICHD	266 (128/138)	NA	Teeth	DBD	NS	Child’s race, sex, parental education, marital status and SES	High
Chiu 2017 [48]	11–14	Italy/PHIME	194 (105/89)	Fe-Mn alloy plant	Teeth	PA, LNMB	Pretnatal T-Mn: ↑: behaviors and motor in boys, T-Mn: ↓: motor (tremor): early postnatal in girls, later postnatal in boys	Children’s age and sex, SES index and tooth attrition	High
do Nascimento 2015 [49]	6–12	Brazil	69 (34/35)	Drinking water from well water	Hair, blood	RCPM	H-Mn: ↓: cognitive function, B-Mn: NS	Age, sex and parents’ education	High
Ericson 2007 [50]	3–9	USA/SECCYD	27 (16/11)	NA	Teeth	FTT, CBCL	Prenatal T-Mn: ↓: behaviors (hyperactivity)	Pb, mothers’ education, income and child ethnicity	High
Frndak 2019 [51]	6–8	Uruguay	345 (155/190)	NA	Hair	CANTAB, W-M	H-Mn: ↑: cognition	Child’s age, sex, Pb, hemoglobin, HOME score, crowding, possessions of wealth and mother’s education	High
Haynes 2015 [52]	7–9	USA/CARES	404 (187/217)	Air-borne Mn from Fe-Mn refinery	Hair, blood	WISC-IV	Both low and high Mn: ↓: IQ	Parent IQ	Medium
Haynes 2018 [7]	7–9	USA/CARES	106 (65/41)	Air-borne Mn from Industry	Hair, blood	WISC-IV	H-Mn: ↓: IQ, B-Mn: NS	Parent IQ	Medium
Hernandez-Bonilla 2011 [53]	7–11	Mexico	172 (84/88)	Air-borne Mn from Mining	Hair, blood	GP, FT, SA	B-Mn: ↓: motor speed and coordination, H-Mn: NS	Pb, Hb, sex, age and maternal education	High
Hernandez-Bonilla 2016 [54]	7–11	Mexico	267 (136/131)	Air-borne Mn from mining district	Hair	ROCF	H-Mn: ↓: visuoperception and short-term visual memory	Pb, Hb, child’s age and sex, motor dexterity and mother’s IQ	High
Horton 2018 [55]	8–11	Mexico/ELEMENT	133 (69/64)	Air pollution and diets	Teeth	BASC-2	Prenatal T-Mn: ↑: behaviors, postnatal T-Mn: ↓: behaviors (internalizing problems)	Maternal education and gestational age	High
Khan 2011 [56]	8–11	Bangladesh	201 (100/101)	Drinking water from well water	Blood	CBCL	B-Mn: NS	Arsenic, sex, BMI, maternal education and arm circumference	High
Kicinski 2015 [57]	13.6–17	Belgium	606 (282/324)	Low-level metal exposure from industrial areas	Blood	FT, CPT, DS	B-Mn: NS	Sex, age, smoking, passive smoking, income, occupation, and maternal education	High
Kim 2009 [58]	8–11	Korea	261 (120/141)	NA	Blood	WISC	B-Mn: ↓: IQ	Age, sex, parental education, income, smoking, birth weight and mother’s age	High
Lucchini 2012a [59]	11–14	Italy	299 (147/152)	Fe-Mn alloy plant	Hair, blood	WISC	NS	Age, sex, BMI, family size, SES, alcohol consumption, area of residence, Hb, ferritin and parity	High
Lucchini 2012b [60]	11–14	Italy	311 (153/158)	Fe-Mn alloy plant	Hair, blood, urine	FT, PA, DPD, LNMB	B-Mn and H-Mn: ↓: motor (tremor), urine, air, water, diet: NS, soil Mn: ↓: tremor intensity	Parity, family size, SES, BMI, maternal education, alcohol intake, smoking, Pb and other metals in air, soil and water.	High
Lucchini 2019 [61]	6–12	Italy	299 (161/138)	Industrial emission, with potential contamination of environment	Hair	WISC, CANTAB	H-Mn: ↓: working memory	Sex, age, maternal IQ and cognitive stimulation besides the confounder distance from the point source	High
Menezes-Filho 2011 [21]	6–12	Brazil	83 (39/44)	Fe-Mn alloy plant	Hair, blood	WISC-III	H-Mn: ↓: cognition, especially in the verbal domain, B-Mn: NS	Maternal education and nutritional status	Medium
Menezes-Filho 2014 [62]	7–12	Brazil	70 (36/34)	Air-borne Mn from Fe-Mn alloy plant	Hair	CBCL	H-Mn: ↓: behaviors (externalizing behaviors), more pronounced in girls	Age, sex and maternal IQ	High
Nascimento 2016 [63]	6–12	Brazil	63 (31/32)	Potential contamination from pesticide	Hair, blood	NEUPSILIN-Inf	B-Mn: ↓: visual attention, visual perception and phonological awareness, H-Mn: ↓: working memory	IQ, age, sex and parents’ education	High
Oulhote 2014 [15]	6–13	Canada	375 (200/175)	Drinking water from ground water	Hair	WASI, CPT-II, FT, SA	H-Mn: ↓: memory, attention, not hyperactivity, motor: a nonlinear association, ↑: 0.3–0.8 μg/g, ↓: > 10 μg/g, but there were very few observations with such high levels	Child’s sex, age, maternal education and IQ, income, maternal depressive symptoms and tap water lead concentrations.	High
Parvez 2011 [64]	8–11	Bangladesh	304 (153/151)	Drinking water from well water	Blood	BOT-2	B-Mn: NS	Sex, school attendance, head circumference, mother’s intelligence, ferritin, selenium and Pb	High
Riojas-Rodríguez 2010 [6]	7–11	Mexico	172 (99/73)	Air-borne Mn from mining district	Hair, blood	WISC	H-Mn: ↓: IQ, B-Mn: NS	Pb, age, sex, nutritional status, maternal education and IQ	High
Rugless 2014 [65]	7–9	USA/CARES	55 (35/20)	Air emissions from Fe-Mn refinery	Hair, blood	APS	H-Mn and B-Mn: ↓: postural balance	Sex, height/weight ratio, parent IQ, education, Pb and age	Medium
Torrente 2005 [66]	12–14	Spain	100 (61/39)	Industrial emission	Hair	AMP	H-Mn: NS	SES and age	Low
Torres-Agustin 2013 [67]	7–11	Mexico	174 (86/88)	Air-borne Mn from mining district	Hair, blood	CAVLT	H-Mn: ↓: long-term memory and learning, B-Mn: NS	Child’s sex, Pb, age, Hb and maternal education	High
Wasserman 2006 [9]	10	Bangladesh	142 (72/70)	Drinking water from well water	Blood	WISC-III	B-Mn: NS	Maternal education and IQ, house type, family ownership of a television, child height and head circumference	High
Wright 2006 [68]	11–13	USA	31 (16/15)	Mining waste	Hair	WASI	H-Mn: ↓: Full Scale IQ, Verbal IQ	Sex and maternal education	Low

^*^: Effects of pollution on neurobehavioral development, and future policies to protect our children; ↓: Negative association; ↑: Positive association; *NA* Not available; *NS* No significant association. *Fe-Mn* Ferro-manganese; *Hb* Hemoglobin; *Mn* Manganese; *Pb* Lead. *B-Mn* Manganese in blood; *H-Mn* Manganese in hair; *T-Mn* Manganese in teeth. *BMI* Body mass index; *HOME score* Home observation for measurement of the environment score; *IQ* Intelligence Quotient; *SES* Socioeconomic status. *CARES* Communities Actively Researching Exposure Study; *ELEMENT* Early Life Exposures in MExico and NeuroToxicology; *NICHD* National Institute of Child Health and Human Development; *PHIME* Public Health Impact of Manganese Exposure in susceptible populations, *SECCYD* Study of Early Child Care and Youth Development

**Table 3:** Neurodevelopmental outcomes of manganese exposure measured in drinking water

Author, Year	Age (Years)	Country/ Study Name	Number (Girls/Boys)	Sources	Neurological Assessments	Associations between Manganese in Drinking Water and Neurodevelopmental Outcomes	Adjustment for Covariates	Study Quality
Neurodevelopmental outcomes from cohort studies								
Dion 2018 [20]	10.5–18	Canada	287 (151/136)	Ground water	WASI	W-Mn and time-averaged W-Mn: ↓: IQ among girls	Maternal IQ, education and income	High
Rahman 2017 [69]	10	Bangladesh	1265 (609/656)	Well water	WISC-IV, SDQ	Prenatal W-Mn: ↑: cognition in girls, W-Mn: ↓: behavior	Maternal IQ, SES, child age, sex, education, height for age, Hb, school type, HOME, tester, number of siblings and arsenic	High
Rodrigues 2016 [70]	1.6–3.3	Bangladesh	525 (264/261)	Drinking water from well water	BSID-III	W-Mn: an inverse-U relationship with fine motor function, cognition: NS	Maternal age, maternal education, passive smoking, child’s sex, HOME score, maternal IQ and child’s hematocrit levels	High
Neurodevelopmental outcomes from cross-sectional studies								
Bouchard 2011 [16]	6–13	Canada	362 (194/168)	Ground water	WASI	W-Mn and estimated Mn intake from water consumption: ↓: IQ, estimated Mn intake from dietary: NS	Maternal education and IQ, income, home stimulation score, family structure, sex and age of child, IQ testing session, source of water and level of iron in tap water	High
Bouchard 2018 [8]	6–14	Canada	259 (132/127)	Drinking water from ground water	WISC-IV	NS, possible beneficial effects in boys	Child’s age, maternal IQ and education, income and IQ tester	High
do Nascimento 2015 [49]	6–12	Brazil	69 (34/35)	Drinking water from well water	RCPM	W-Mn: ↓: cognitive function	Age, sex and parents’ education	High
Khan 2011 [56]	8–11	Bangladesh	201 (100/101)	Drinking water from well water	CBCL	W-Mn: ↓: behaviors (classroom behavioral problems)	Arsenic, sex, BMI, maternal education and arm circumference	High
Khan 2012 [71]	8–11	Bangladesh	840 (444/396)	Well water	AARES	W-Mn > 400 μg/L: ↓: mathematics test	School-grade, parental education and head circumference and controlling for within-teacher correlations in rating the children	High
Nascimento 2016 [63]	6–12	Brazil	63 (31/32)	Potential contamination from pesticide	NEUPSILIN-Inf	W-Mn: ↓: written language and executive functions	IQ, age, sex and parents’ education	High
Oulhote 2014 [15]	6–13	Canada	375 (200/175)	Drinking water from ground water	WASI, CPT-II, FT, SA	W-Mn: ↓: memory, Mn intake from water:↓: motor function	Child’s sex, age, maternal education and IQ, income, maternal depressive symptoms and tap water lead	High
Wasserman 2006 [9]	10	Bangladesh	142 (72/70)	Drinking water from well water	WISC-III	W-Mn: ↓: IQ	Maternal education and IQ, house type, family ownership of a television, child height and head circumference	High

↓: Negative association; ↑: Positive association; *NS* No significant association. *Hb* Hemoglobin; *Mn* Manganese. *W-Mn* Manganese in drinking water. *BMI* Body mass index; *HOME score* Home observation for measurement of the environment score; *IQ* Intelligence Quotient; *SES* Socioeconomic status

The neurological outcomes were assessed more frequently among children between 6 and 18 years of age than children under 6 years old. Amongst children under 6 years old, most studies were cohort studies with the different editions of Bayley Scales of Infant and Toddler Development applied to assess neurodevelopment, and the measurements of manganese mainly reflected prenatal exposures. In the older groups, the well-defined versions of Wechsler Intelligence Scale for Children were used to assess the children’s general cognitive abilities. Specific cognitive functions were assessed through its subtests. For behavioral performance, the variant editions of Conners’ Rating Scale were applied in most studies. For motor coordination, Finger Tapping Test and Luria Nebraska Motor Battery were administered in most studies. Among these studies, the most adjusted confounders in the mathematical model were maternal education, maternal intelligence, child age and sex, which were selected based on established or plausible associations with neurodevelopment. A large percentage (44/55) of included studies was of high quality (see Additional file 2). Except for three studies, all the others described the efforts to address potential sources of bias, such as blinding of exposure status and outcomes assessment, using validated assessment scales and previously trained psychologists.

### Manganese in biomarkers and neurodevelopmental outcomes

In children under 6 years of age, evidence from cohort studies in Table 1 revealed that higher manganese exposure had a negative effect on neurodevelopment [29–36], mainly cognitive and motor development. These studies enrolled pregnant women and mainly collected biomarker tissues, such as cord blood, maternal blood and hair, as well as placenta at delivery [29, 30, 32, 34–36]. One study sampled maternal hair and blood at intervals 1–3 times during pregnancy [33]. These biomarkers mentioned above were used to indicate prenatal exposure. The other study collected shed teeth from children beginning at age 7 [31], which provides fine scale temporal profiles of Mn concentrations over the prenatal and early childhood periods. Neurodevelopmental outcomes were assessed by trained psychometricians at follow-up, mainly at 1–2 years of age.

The other two birth cohort studies found an inverted U-shaped association between manganese exposure and cognitive or motor development [27, 28] (see Table 1). Claus Henn et al. (2010) reported that the effect of manganese was apparent for 12-month but diminished for mental development scores at older ages [28], suggesting the possible existence of critical developmental windows. Chung et al. (2015) found a nonlinear dose-response relationship between maternal blood manganese at term and 6-month psychomotor development scores, with a peak point approximately 24–28 μg/L, suggesting adverse neurodevelopmental effects of both low (< 20.0 μg/L) and high (≥ 30.0 μg/L) maternal blood manganese concentrations [27].

The results from cohort studies concerning children over 6 years old were intriguing. Two follow-up studies in Bangladesh and Canada were conducted to evaluate whether changes in drinking water manganese exposure were associated with changes in child intellectual outcomes. In Bangladesh, Wasserman et al. (2016) found that during 2 years of follow-up, the reduction in exposure (indicated by manganese in blood, B-Mn*)* was not, for the most part, translated into improvements in child IQ. In this cohort, baseline B-Mn was negatively associated with working memory after covariate adjustment [19]. In Quebec (Canada), the result revealed that, for children whose Mn concentrations in their water supply increased between baseline and follow-up, their Performance IQ scores decreased significantly. On the other hand, at follow-up, higher manganese in drinking water was associated with lower Performance IQ in girls, whereas the opposite was observed in boys. Similar trends were observed in hair [20]. Although the results of cohort studies need to be verified, they also suggest the importance of preventing such exposures.

Inconsistent conclusions were drawn from three cohort studies, one measured manganese in cord blood and spot urine [39], the other two sampled dentine of incisors [37, 38] (see Table 1). The birth cohort study in China reported that urinary Mn concentrations, but not cord blood manganese, were positively associated with Performance IQ of school-age children, especially in girls [39]. Mora et al. (2015) found that higher prenatal and early postnatal manganese in teeth (T-Mn) were adversely associated with behavioral outcomes, namely internalizing, externalizing and hyperactivity problems, in children at 7 and 10.5 years. In the sex stratified models, Mora et al.(2015) found that higher prenatal and postnatal T-Mn were associated with better memory abilities at ages 9 and 10.5, and better cognitive and motor outcomes at ages 7 and 10.5 years, among boys only [38]. On the other hand, Claus Henn et al. (2018) found that higher postnatal T-Mn was negatively associated with both Wide Range Assessment of Visual Motor Abilities (WRAVMA) total and visual spatial subtest scores, among boys only [37]. Mn interactions with lead (Pb) were also examined. Mora et al. (2015) reported that higher prenatal T-Mn was associated with poorer visuospatial memory outcomes at 9 years and worse cognitive scores at 7 and 10.5 years in children with higher prenatal blood lead concentrations (≥ 0.8 μg/dL) [38]. And Claus Henn et al. (2018) found that tooth Mn was positively associated with visual spatial and total WRAVMA scores in the second trimester, among children with lower (< median) tooth Pb concentrations, while no significant Mn association was observed at high Pb concentrations [37]. These inconsistent findings may be due to differences in biomarkers (blood and urine vs. teeth) or sources of Mn exposure (Mn-containing fungicides vs. dietary and airborne sources).

Although most cohort studies found adverse association between manganese exposure and neurodevelopment, Mn interactions with sex and other metals, such as Pb, were gaining attention. Among these studies, only six studies described the sources of manganese exposure during pregancy, such as mining, mancozeb and drinking water, the concentration of manganese was only measured in drinking water in two studies [19, 20]. And Dion et al. (2018) reported that Mn in hair (H-Mn) correlated with W-Mn at follow-up (*r*, 0.48; *p* <  0.001) and with time-averaged W-Mn (*r*, 0.43; *p* <  0.001) [20].

Two out of the 37 cross-sectional studies investigated associations between Mn exposure and developmental scores in infants. Postnatal manganese exposure were measured in breast milk, blood, urine and hair, no significant association was observed [40, 41], with significantly negative association in the unadjusted model [41] (see Table 2).

There were 35 studies concerning children over 6 years old, and these also measured manganese in related biomarkers, such as hair (*n* = 24) [6–8, 15, 16, 21, 22, 43, 45, 46, 49, 51–54, 59–63, 65–68], blood (*n* = 17) [6, 7, 9, 21, 44, 49, 52, 53, 56–60, 63–65, 67], teeth (*n* = 5) [42, 47, 48, 50, 55], saliva, toe nail [8] and urine [60]. One study could be included when using more than one biomarker, as was the case in New Brunswick (Canada), which measured manganese in hair, saliva and toe nail [8].

A central result was that elevated H-Mn was associated with poorer cognitive and behavioral performance in most studies (*n* = 17), in terms of IQ [6, 7, 16, 21, 22, 43, 49, 52, 68], working memory [61, 63], verbal memory [46], visuoperception and short-term visual memory [54], long-term memory and learning abilities [67], memory and attention [15], hyperactivity behaviors [45, 46], oppositional behaviors [45] and externalizing behavioral problems [62] (see Table 2). Among them, Oulhote et al. (2014) found that there was no significant association between manganese exposure and hyperactivity [15]. In this case, a large percent (13/17) of the studies reported that the mean of manganese in hair exceeded 0.55 μg/g, which was similar to the mean concentration from control groups [6, 54]. Haynes et al. (2015) also found that compared with the middle two quartiles, the lowest quartiles of H-Mn (< 0.21 μg/g) was associated with significantly lower mean perceptual reasoning scores [52]. Similarly, one cross-sectional study revealed a positive association between H-Mn and cognitive function in children aged at 6–8 years, with a low median concentration of Mn in hair (0.82 ng/g) [51]. No significant associations were found in three studies in terms of cognitive functions [8, 59, 66] and behavioral performance [59], with the average of manganese in hair ranged from 0.17 μg/g to 0.3 μg/g. For motor function, two studies showed that elevated H-Mn was associated with tremor intensity [60] and poor postural balance [65], while two articles found no association between H-Mn and motor function [46, 53]. In another publication, Oulhote et al. (2014) found a nonlinear association between H-Mn and motor function, with a slight increase at concentrations between 0.3 and 0.8 μg/g, and an apparent decrease in scores at H-Mn >  10 μg/g, although there were very few observations with such high concentrations [15]. This inconsistency may possibly due to the different levels of manganese exposure and the sensitivity of scales, as the average concentration of H-Mn ranged widely from 0.16 μg/g to 14.6 μg/g [15, 46, 53, 60, 65]. Carvalho et al. (2018) reported poorer cognition and behavior, while no effect on motor in the same exposure population [46] (see Table 2).

In contrast to hair, most (*n* = 9) reports in Table 2 indicated that B-Mn was non-significantly associated with cognitive and behavioral development [6, 7, 9, 21, 49, 56, 57, 59, 67]. However, two studies did show that elevated B-Mn was associated with poorer cognitive development, when using IQ [58], visual attention, visual perception and phonological awareness [63] as outcome measures. Two publications suggested that both low and high B-Mn were negatively associated with cognitive and behavioral development [44, 52]. In relation to motor development, three studies showed that elevated B-Mn was associated with impairment of motor functions, namely tremor intensity [60], postural balance [65], coordination and motor speed [53]. However, one study indicated no significant association [64] (see Table 2). Among these reports, the mean concentration of manganese in blood was mainly around 10 μg/L, suggesting the relatively homeostatic regulation of blood manganese.

Five publications used teeth as a biomarker [42, 47, 48, 50, 55] (see Table 2). One study found an inverted U-shaped association between prenatal Mn and visuospatial ability in girls, no significant associations were found in postnatal Mn [42]. Ericson et al. (2007) found that higher Mn in teeth was adversely associated with behavioral outcomes [50]. Horton et al.(2018) revealed that prenatal Mn exposure appeared to be protective against behavioral outcomes, yet postnatal Mn appeared as a risk factor for behavioral outcomes [55]. Two of which indicated that there were no significant associations between Mn in deciduous teeth and behavioral [47] and motor development [48]. These results suggested that Mn associations were partly driven by exposure timing and modified by sex. Three studies also found that tooth Mn concentrations were higher in the prenatal than postnatal period [42, 48, 55], indicating a greater demand for manganese in the prenatal period. No significant associations were observed between neurodevelopmental outcomes and Mn in saliva, toe nail [8], and urine [60] (see Table 2).

### Manganese in drinking water and neurodevelopmental outcomes

Evidience from cohort studies indicated that elevated W-Mn was associated with lower IQ scores in girls [20] and the increased risk of children’s behavioral problems at 10 years of age [69] (see Table 3). While Rodrigues et al. (2016) also found an inverted U-shaped association between W-Mn and motor development with an inflection point around 400 μg/L [70].

Most (*n* = 7) cross-sectional studies found that higher W-Mn was associated with poorer cognitive and behavioral function, such as IQ [9, 16, 49], memory [15], written language [63], mathematics scores [71] and the risk of behavioral problems [56]. The mean concentrations of W-Mn were shown to range from 795 to 1387.9 μg/L in three studies conducted in Araihazar, a rural area of Bangladesh [9, 56, 71], which were much higher than W-Mn in Canada, with its arithmetic mean of 98 μg/L reported in two studies [15, 16]. The W-Mn in two studies conducted in Brazil was much lower, with the mean of W-Mn around 20 μg/L in the rural group [49, 63]. By contrast, two reports found no clear association between W-Mn and childhood IQ [8] and behavioral function [15] (see Table 3). Both studies were conducted in Canada [8, 15], one described a situation where W-Mn was low, with approximately half of children’s home tap water with a manganese concentration less than 5 μg/L [8]. For motor function, the association with W-Mn was significant, with a threshold indicating that scores decreased more steeply at concentrations above 180 μg/L, and this research also found that manganese intake from water was negatively associated with motor function [15]. Of note, most studies also measured manganese in hair or blood, the conclusions were mainly consistent with H-Mn [15, 16, 49, 63], whereas the inconsistency was observed in blood [9, 56].

### Pooled effect estimates for IQ scores

The details for our meta-analysis were extracted to include three manganese exposure metrics: hair, blood and drinking water, as shown in Additional file 3. Among these studies, seven researches had a cross-sectional design, two cohort studies were treated as cross-sectional studies by using the associations between Mn exposure and concurrent IQ scores at baseline examinations [19] or follow-up examinations [20].

**Fig. 2:**
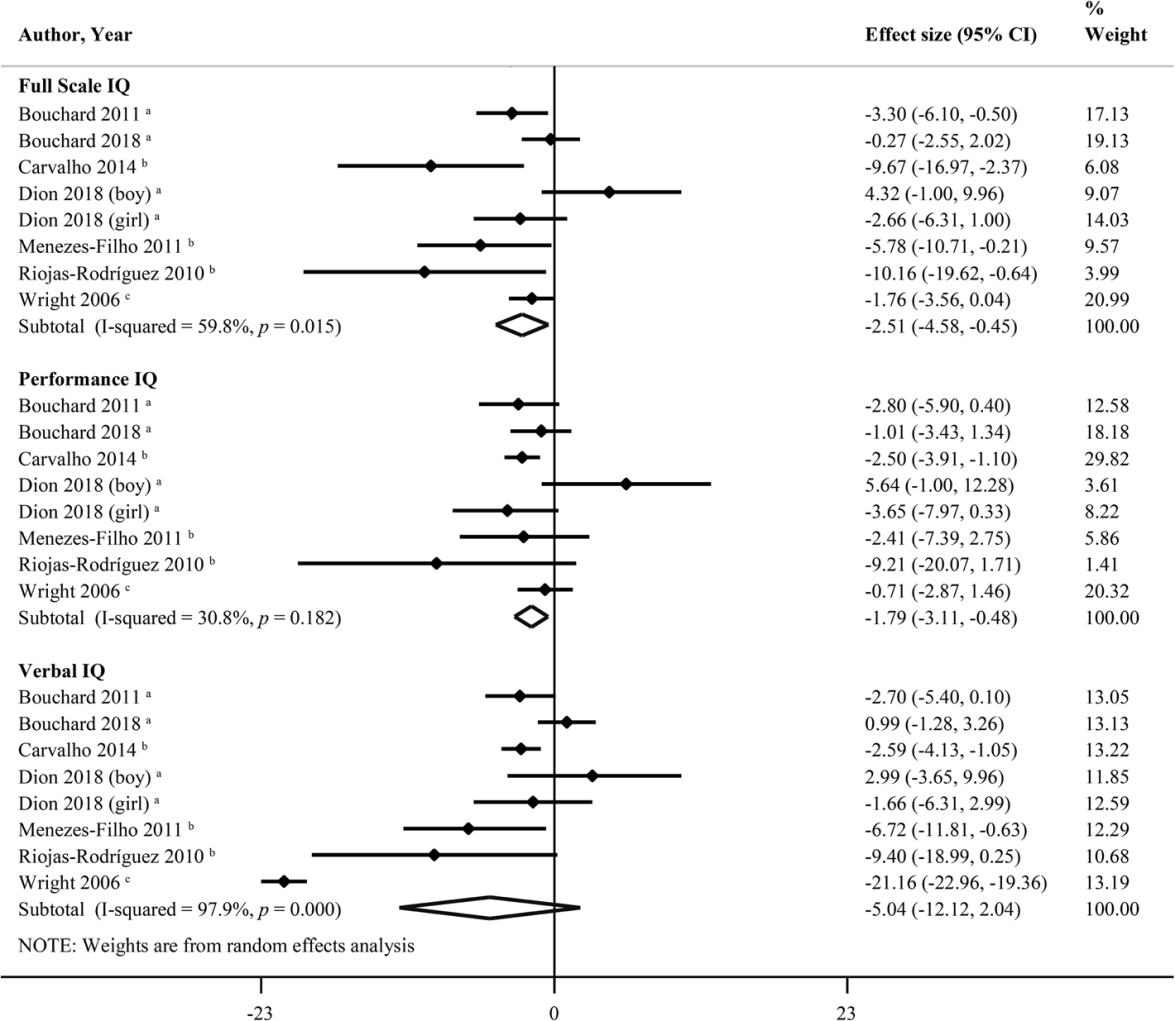
Forest plots of effect size on intellectual quotient (IQ) by a 10-fold increase in hair manganese. a: waterborne manganese exposure, b: airborne manganese exposure, c: manganese exposure from mining waste

Figure 2 clearly shows that a 10-fold increase in hair manganese is associated with a decrease of 2.51 points (95% CI, − 4.58, − 0.45; *I*^*2*^ = 59.8%) in Full Scale IQ of children aged 6–18 years. Of note, this inverse relationship remained significant when we conducted a sensitivity analysis in which one study was removed at a time (see Additional file 4). The pooled results with respect to Performance IQ were not extremely robust and should be investigated further. Heterogeneity was 59.8% for Full Scale IQ, as a “leave-one-out” analysis revealed that there still existed some heterogeneities. Next, we performed a subgroup analysis based on the source of exposure, revealing a significantly inverse association between H-Mn and IQ scores from airborne manganese exposure [6, 21, 22], but not from waterborne [8, 16, 20] or mining waste manganese exposure [68]. The pooled *β* for the 10-fold increase in hair manganese from airborne manganese exposure was associated with a decrease of 7.62 points (95% CI, − 11.51, − 3.73; *I*^*2*^ = 0%) for Full Scale IQ. For Performance IQ, the decrease would be 2.60 points (95% CI, − 3.94, − 1.25; *I*^*2*^ = 0%), and 4.56 points decrease (95% CI, − 8.33, − 0.79; *I*^*2*^ = 45.5%) for Verbal IQ. Unexpectedly, the concentrations of H-Mn from airborne manganese exposure were much higher than the others. Therefore, we concluded that both the source of manganese exposure and the concentrations of H-Mn likely account, at least in part, for this relatively high heterogeneity. The results from Begg’s and Egger’s tests did not suggest the existence of publication bias.

The meta-analysis in drinking water and blood revealed no significant effects (Additional files 5, 6). Of significance, among the reports that used both hair and blood as biomarkers, a large percent (7/11) of which indicated that H-Mn, but not B-Mn, was negatively associated with cognitive development [6, 7, 21, 49, 52, 59, 67] (Table 2).

### Different biomarkers in Mn exposure and neurodevelopment

In the elder group, most studies used hair and blood as biomarkers. While teeth was also used as a biomarker in some publications with inconsistent Mn associations. Some researches also measured manganese in environmental samples, such as drinkable water, soil and particles. The correlations between manganese in drinking water and biomarker (hair or blood) or different biomarkers were analyzed in nine studies, as shown in Additional file 7. Among these publications, six studies used Spearman’s rank correlation [7, 9, 49, 56, 63, 67], as the distributions of manganese concentrations in biomarkers and drinking water were considerably skewed. Three studies analyzed the correlation using Pearson correlation tests, among these studies, the concentrations of manganese in indicators were transformed in order to make distributions more symmetrical for Pearson correlation tests [20, 21, 52].

The preliminary meta-analysis was conducted to gain a pooled result of correlations between different manganese indicators (see Additional file 7). The correlation between H-Mn and W-Mn indicated that they did have significance, and the pooled correlation coefficient *r* was 0.48 (95% CI, 0.40, 0.55). By contrast, the summary correlation between B-Mn and W-Mn, even B-Mn and H-Mn indicated that there had no significance. Although different analytical methods were applied in three studies that analyzed the correlation between H-Mn and W-Mn, the conclusion was consistent [20, 49, 63].

Wasserman et al. (2011) found that blood did not vary predictably across the low and high W-Mn groups, suggesting that blood may not be a good reflection of drinking water Mn exposure [72]. From the airborne manganese exposure, Torres-Agustin et al. (2013) observed a statistically significant difference between the two groups in the median blood Mn concentrations of 8.0 and 9.5 μg/L for non-exposed and exposed children, respectively. Meanwhile, hair Mn concentrations in exposed children were, on average, 20 times higher (median 12.6 and mean 14.2 μg/g) than the nonexposed group (median 0.6 and mean 0.73 μg/g) [67]. These results indicate that hair is more sensitive than blood to reflect environmental manganese exposure.

In infants, one report found that maternal and cord blood manganese concentrations were correlated, though in a nonlinear manner. Here, the median manganese in cord blood was nearly twice the median concentration in maternal blood, unexpectedly, the inverse associations were found between manganese in maternal blood, but not cord blood, and early childhood mental and psychomotor development scores [29]. The other biomarkers (i.e. maternal and infant hair and placenta) used to reflect prenatal Mn exposure did not show the correlation.

### Sex specific exposure-response relationships

Evidence from eight cohort studies yielded inconsistent conclusions as to sexual effects (see Table 4). Three of four studies reported a statistically significant sex interaction (*p* <  0.05), and concluded that girls were more susceptible to manganese exposure than boys in terms of cognition and motor [20, 31, 33]. While Takser et al. (2003) found that in children at 3 years, the hand skill score was negatively associated with cord blood Mn in boys (*p* = 0.002), but not in girls [34]. Two cohort studies found a positive non-statistically significant association between manganese exposure and cognitive development in girls [69] and cognitive and motor development in boys [38]. Sex interaction *p*-values in the remaining two reports were not available. Positive association between urinary Mn concentrations and Performance IQ of children was observed, especially in girls [39]. And Claus Henn et al. (2018) found significantly negative associations between T-Mn and visual spatial score, among boys only [37].

**Table 4:** Characteristics of the 18 studies that conducted sex-stratified analyses

Author, Year	Age (Years)	Neurodevelopment	Effect on Boys	Effect on Girls	*p*-value of Interaction	Manganese Concentrations
Neurodevelopmental outcomes from cohort studies						
Claus Henn 2018 [37]	6–16	Cognition	↓	NS	NA	Teeth: 12^ab^ (*n* = 138)
Dion 2018 [20]	10.5–18	Cognition	↑ (W-Mn)	↓ (W-Mn)	< 0.01 (W-Mn)	Drinking water: 14.5 μg/L^c^ (*n* = 287), hair: 1.4 μg/g^c^ (*n* = 274)
Gunier 2015 [31]	0.5, 1, 2	Cognition and motor	NS	↓	0.02 (Cognition), 0.03 (Motor)	Teeth: prenatal: 0.51 ± 0.19^bd^ (*n* = 197), postnatal: 0.20 ± 0.23^bd^ (*n* = 193)
Mora 2015 [38]	7, 9, 10.5	Cognition and motor	↑	NS	< 0.1	Teeth: prenatal: 0.50 ± 0.18^bd^ (*n* = 248), postnatal: 0.19 ± 0.21^bd^ (*n* = 244)
Mora 2018 [33]	1	Cognition	NS	↓	0.01	Maternal hair: 3.7 ± 5.4 μg/g^d^ (*n* = 661), maternal blood: 24.4 ± 6.2 μg/L^d^ (*n* = 571)
Rahman 2017 [69]	10	Cognition	NS	↑	< 0.081	Drinking water: 339 μg/L^a^ (*n* = 1265)
Takser 2003 [34]	Newborns	Motor	↓	NS	0.03	Cord blood: 38.5 μg/L^c^ (*n* = 222), maternal blood: 20.4 μg/L^c^ (*n* = 222), maternal hair: 0.36 μg/g^c^ (*n* = 173), newborns hair: 0.75 μg/g^c^ (*n* = 173), placenta: 0.1 μg/g^c^ (*n* = 200)
Zhou 2019 [39]	6–8	Cognition	↑	NS	NA	Cord blood: 29.29 ± 1.48 μg/L (*n* = 296), urine: 0.66 ± 3.81 μg/L (*n* = 207)
Neurodevelopmental outcomes from cross-sectional studies						
Bauer 2017 [42]	10–14	Cognition	NS	↓	0.05	Teeth: prenatal: 0.42^cb^ (*n* = 142), postnatal: 0.12^cb^ (*n* = 142)
Bouchard 2011 [16]	6–13	Cognition	NS	↓	0.55 (H-Mn), 0.14 (W-Mn)	Hair: 0.7 μg/g^a^ (*n* = 302), drinking water: 0.8 μg/L^a^ (*n* = 362)
Bouchard 2018 [8]	6–14	Cognition	NS (Toe nail), ↑ (W-Mn)	↓ (Toe nail), NS (W-Mn)	0.028 (Toe nail), 0.015 (W-Mn)	Toe nail: 2.0 μg/g^c^ (*n* = 258), hair: 0.3 μg/g^c^ (*n* = 258), saliva: 1.1 μg/L^c^ (*n* = 226), drinking water: 5.2/7.3 μg/L^c^ (boy: 127, girl: 132)
Carvalho 2018 [46]	7–12	Cognition	↓	NS	0.047	Hair: 11.5 μg/g^a^ (*n* = 70)
Chiu 2017 [48]	11–14	Motor	NS (Prenatal and postnatal), ↓ (Childhood)	↓ (Prenatal and postnatal), NS (Childhood)	< 0.05 (Prenatal), < 0.01 (Postnatal), 0.01 (Childhood)	Teeth: prenatal: 0.43^ab^ (*n* = 189), postnatal: 0.13^ab^ (*n* = 185)
Hernandez-Bonilla 2016 [54]	7–11	Cognition	NS	↓	< 0.15	Hair: control: 0.55 μg/g^c^ (*n* = 119), exposed: 5.25 μg/g^c^ (*n* = 148)
Menezes-Filhoet 2014 [62]	7–12	Behavior	NS	↓	NA	Hair: boys: 12.1 μg/g^a^ (*n* = 34), girls: 12.4 μg/g ^a^ (*n* = 36)
Rink 2014 [41]	1.1–3.7	Cognition	↑	NS	< 0.05	Hair: 0.98 ± 0.74 μg/g^d^ (*n* = 60)
Riojas-Rodríguez 2010 [6]	7–11	Cognition	NS	↓	NA	Control: hair: 0.57 μg/g, blood: 8.22 μg/L^c^ (*n* = 93), exposed: hair: 12.13 μg/g, blood: 9.71 μg/L^c^ (*n* = 79)
Torres-Agustinet 2013 [67]	7–11	Cognition	NS	↓	NA	Control: hair: 0.6 μg/g, blood: 8.0 μg/L^a^ (*n* = 95), exposed: hair: 12.6 μg/g, blood:9.5 μg/L^a^ (*n* = 79)

↓: Negative association; ↑: Positive association; *NA* Not available; *NS* No significant association. a: Median; b: ^55^Mn: ^43^Ca the area under the curve (AUC) × 10^4^; c: Geometric mean; d: Mean ± standard deviation. *H-Mn* Manganese in hair; *W-Mn* Manganese in drinking water

Seven cross-sectional studies consistently concluded that girls were more susceptible to manganese exposure than boys with respect to cognition and behavior [6, 8, 16, 42, 54, 62, 67], with non-statistically significant association in three reports [16, 42, 54] and interaction *p*-values not available in four studies [6, 8, 62, 67]. Three studies reported a statistically significant interaction between manganese exposure and sex (*p* <  0.05), without a clear pattern [41, 46, 48]. Rink et al. (2014) found that in children aged 14–45 months, H-Mn was negatively associated with the cognitive, receptive language and expressive language scores for girls only in the unadjusted model [41]. While negative association between H-Mn and the free recall after interference score was observed, especially in boys [46]. Chiu et al. (2017) found that higher prenatal Mn was associated with better body stability in boys, with opposite associations in girls. For tremor, on the other hand, higher early postnatal Mn was associated with increased right-hand center frequency in girls, but increased Mn concentration at the later postnatal period was associated with increased center frequency in boys [48].

**Fig. 3:**
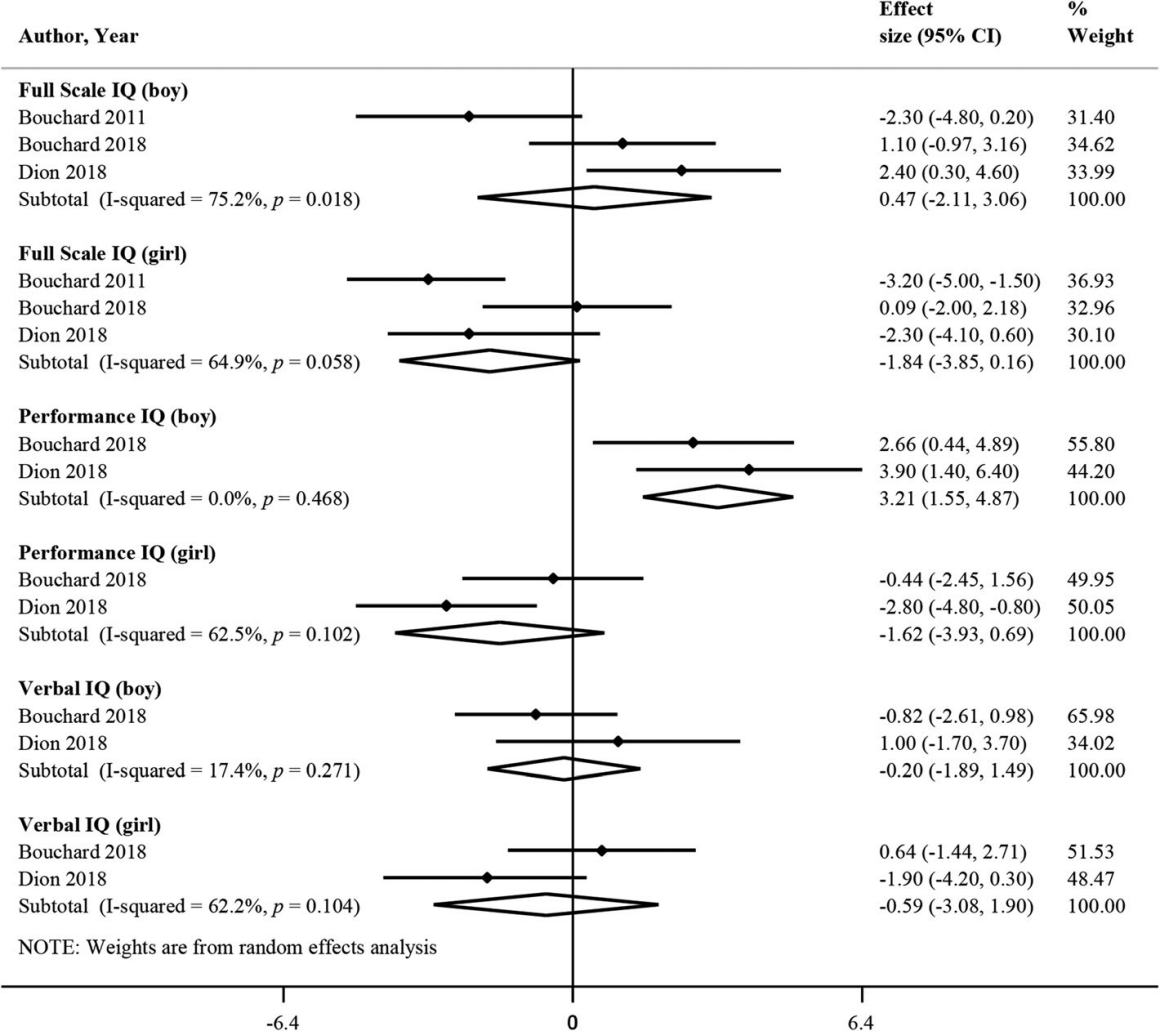
Meta-analysis of studies that stratified by sex reporting the effect of a 10-fold increase in drinking water manganese on intellectual quotient (IQ)

Three studies met the criteria for meta-analysis since they had a similar number of participants, and all adjusted for potential confounders such as maternal nonverbal intelligence, maternal education and family income [8, 16, 20]. Bouchard et al. (2011) only provided the detail of sex-stratified analysis for Full Scale IQ [16], therefore, there were two studies in the meta-analysis for Performance IQ and Verbal IQ. Figure 3 presents that higher W-Mn is associated with better Performance IQ among boys only (change in scores for a 10-fold increase in concentration, *β* = 3.21; 95% CI, 1.55, 4.87), in these two studies, a large percentage of children were exposed to drinking water manganese under 50 μg/L (the esthetic Canadian guideline concentration for W-Mn) [8, 20]. The meta-analysis on 3 studies concerning childhood IQ and H-Mn, found no significant difference between boys and girls [8, 16, 20] (Additional file 8).

## Discussion

This systemic review and meta-analysis was based on 55 studies, including 17 cohort studies and 38 cross-sectional studies, with 13,388 participants. Evidence from cohort studies found that higher manganese exposure had a negative effect on neurodevelopment, mainly cognitive and motor skills in children under 6 years of age. In children aged 6–18 years old, results from cross-sectional studies revealed that higher H-Mn and W-Mn, but not B-Mn or T-Mn, were negatively associated with cognitive and behavioral performance. Of these cross-sectional studies, most studies reported that the mean of manganese in hair was more than 0.55 μg/g. The pooled results in H-Mn revealed that a 10-fold increase in H-Mn was associated with a decrease of 2.51 points (95% CI, − 4.58, − 0.45) in Full Scale IQ in children aged 6–18 years old. In the elder group, hair was the most consistent and reliable indicator of manganese exposure. These published data did demonstrate sex differences upon manganese exposure, without a clear pattern, possibly girls were more susceptible to manganese exposure than boys.

It is worth noting that the association between manganese exposure and motor performance was inconsistent in hair, blood and teeth. Only the results from infants [27, 34, 36, 70] and one study from the elder group that measured manganese in drinking water [15] supported the conclusion that higher manganese exposure had a negative effect on motor skills. It is a fact that occupational manganese exposure in adults can cause parkinsonian-like movement disorders [12]. Of note, animal studies reported that the increased brain manganese concentrations, either by Mn exposure or genetic strategies can cause severe motor deficits [73–75]. This consequence of excess Mn appears to be partly due to its interaction with other metals, like iron, operating through the post-transcriptional iron-responsive element driven regulatory mechanisms [76, 77]. Future studies are needed to evaluate the association between manganese exposure and motor performance in children.

Given the emerging evidence associating elevated Mn exposure with neurological impairments in children, it is critical to explore children’s exposure to Mn from the different sources. Evidence from cross-sectional studies indicated that groundwater and industrial emissions from ferromanganese alloy plants and mining were the main sources of environmental manganese exposure. Therefore, children were exposed to manganese mainly by inhaling pollutants from industrial emissions and by drinking water. Compared to cross-sectional studies (Table 2), most birth cohort studies enrolled mother-infant pairs in hospitals or clinics without specific source of manganese exposure. Among these studies, they all measured manganese in biomarkers, which can reflect all sources (i.e. diet, air and water) and routes of exposures [78], although Mn homeostasis differs markedly for dietary uptake and inhalation. Additionally, almost all these studies carried out the analysis based on percentile grouping, with some studies yielding additional indication of a dose-response relation of any shape (i.e. linear or an inverted U). Nevertheless, further research is needed to explore the mechanism with respect to the absorption and distribution of different sources of manganese.

Additionally, there is a particular need for a consistent biomarker to accurately assess children’s exposure to Mn. The concentrations of manganese were frequently measured in hair and blood to reflect internal Mn dose in children aged 6–18 years. Results from water-borne and air-borne manganese exposure indicated that hair was more sensitive than blood to reflect the load of manganese in the body. What is more, hair manganese from airborne manganese exposure was much higher than waterborne manganese exposure and was negatively associated with childhood IQ scores.

From these analyses, hair is the more promising measure of long term Mn exposures when compared to blood (with a half-life of 4 or 39 days due to the different elimination pathways [79]). Many metals are deposited in keratin, a component of hair, and the relatively slow growth rate of hair means that hair represents integrated exposures [80]. The 2 cm of newly grown hair was used for measuring the concentration of manganese in most publications, which reflects the exposure during the 2–4 months before sampling [81]. In addition, teeth also reflects long-term exposures as a slow metabolism and accumulation of Mn occurs in teeth [82]. Among eight studies that used teeth as a biomarker, all of these studies measured manganese in naturally shed deciduous milk teeth, while the teeth type varied among these studies, such as for incisors, canines and molars. In most studies, cumulative Mn exposures were estimated in incisors that were free of obvious defects such as caries and extensive tooth wear, which reflect manganese exposure from 13 to 16 weeks after gestation to approximately 1 year of age [83]. In fact, animal studies showed that H-Mn was significantly correlated with T-Mn. Furthermore, correlation coefficients clearly supported links between H-Mn and cognitive functions, reflected by escape latencies and number of platform crossings, and the correlations were better than those in teeth [84]. Additionally, hair is easier to obtain than teeth. Toe-nail can also be employed as a tissue source to measure of chronic exposure to this metal [8], but this technique has rarely been used when establishing environmental manganese exposures in children. The characteristics of relevant biomarkers are summarized in Table 5 [79, 81–83, 85–99].

**Table 5:** Characteristics of relevant biomarkers used in children

Biomarkers	Characteristics	Advantages	Limitations
Hair	Reflects the exposure during the 2–4 months before sampling [81]	Easy to collect, store and manipulate, non-invasive, most consistent and valid biomarker in pediatric epidemiology [85]	Pigmentation and potential external contamination [86]
Blood	With a half-life of 4 or 39 days due to different elimination pathways [79]	Obtained easily and less influence of external contamination [87]	Correlated poorly with exposure [88]
Teeth	Reflects the exposure from 13 to 16 weeks after gestation to approximately one year of age [83]	Non-invasive, provides precise exposure information, distinguishes the prenatal and postnatal exposure [82]	Caries and teeth with attrition contained less metal [89], relatively difficult to obtain and measure
Saliva and urine	secretes 0.8 to 1.5 L of saliva each day, only a small fraction of Mn eliminates in urine [90]	Non-invasive and easy to collect [90]	Correlated poorly with exposure [88, 91, 92], fairly large variation [93]
Toe nail	Reflects an exposure of 7–12 months before sampling [94, 95]	Easy collection, storage and transport [96], correlated with exposure [91]	Difficult to collect sufficient toenail from infants and potential external contamination [97]
Cord blood	Reflects an exposure of the last trimester [97]	Correlated with manganese in dentin [98]	Not feasible to obtain at different stages of pregnancy [98]
Maternal blood	Mn enters the fetus via an active transport mechanism [99]	Readily sampled [98]	Maternal Mn biomarkers may not accurately reflect Mn levels in fetal tissues [98]

*Mn* Manganese

Overall, this review suggests that hair is the most reliable indicator of environmental manganese exposure in children aged 6–18 years old. Traditionally, the main problem of using hair as a biomarker is the potential for external contamination. In response to this, except for a study published in 2007 [45], all other studies that measured manganese in hair had used defined cleaning methodologies to eliminate external contamination. Eastman et al. (2013), for example, developed a hair cleaning methodology to effectively eliminate exogenous metal contamination [86]. This method can substantiate the use of hair as a biomarker of environmental Mn exposure in children. It should be noted that hair dye or other topical treatment could influence the content of manganese in hair [100], although topical hair treatment is unfrequent in children. In spite of this, two studies, now included, also excluded children who reported using hair dye in the preceding 5 months [15, 16]. Further work is needed to determine the utility of hair as a biomarker in preschoolers exposed to manganese. For infants, there appears to be insufficient hair to be analyzed. It is worth noting that teeth provides integrated measures of exposure over the prenatal and early childhood periods of their development, perhaps presenting as a promising biomarker of manganese exposure in infants.

It is always important to accurately determine the safe range of manganese exposure. For this reason, we extracted the reference range or cut-off point used in the reviewed articles (see Additional file 9), while limitations in our data precluded us from directly addressing some aspects of this important issue. In regard to H-Mn, we found that the cut-off point was much higher than the upper limit value of reference range. Accordingly, negative associations between H-Mn and neurodevelopment were observed in two studies that used 2 or 3 μg/g as the cut-off point [43, 45]. And Haynes et al. (2015) found that compared with 0.21–0.75 μg/g, both lower and higher H-Mn were associated with lower IQ scores [52], this may be closer to the possible reference range in children.

It will be critical to consider the timing of Mn exposures, because there may be certain sensitive periods to the effects of environmental manganese exposures in the developing brain. Takser et al. (2003) found that there were negative relationships between cord blood Mn concentrations and several psychomotor sub-scales at age of 3 years, but not at 9 months or 6 years, after adjustment for potential confounders [34].

Some included studies measured prenatal exposure, as indicated by manganese in maternal and cord blood, maternal and infant hair and placenta. Other studies measured manganese in teeth, which reflects prenatal and postnatal exposure (from 13 to 16 weeks after gestation to 1 year of age)*.* Most of the included studies measured postnatal manganese exposure with a cross-sectional design. Hair was the frequently-used biomarker, where that analyzed 2 cm closest to the scalp reflects the exposure during the 2–4 months before sampling [81]. Among these studies, we tend to believe that manganese exposure is continuous, as some cross-sectional studies recruited children who had lived in the same community for a minimum of 3 months or 5 years, to ensure continuous exposure to the same source for this period of time. The follow-up study is warranted to explore the periods of critical vulnerability of environmental manganese exposures.

In this review, information regarding sex differences of manganese exposure from both cohort studies and cross-sectional studies were inconsistent. Perhaps there was a trend showing that girls were more susceptible to manganese exposure than boys. While almost all studies found no significant sex differences for Mn in biomarkers and drinking water, except for four studies without the relevant details available [34, 39, 54, 67]. Given that most studies were not specifically designed to evaluate sex-interactions, therefore, low statistical power may in part explain some of the inconsistency between studies.

Recently a study of single nucleotide polymorphisms in Mn transporter genes *SLC30A10* and *SLC39A8* also found a sex difference between Mn concentrations and genotypes [101]. The mechanisms behind potential sex differences in Mn toxicity are complicated, possibly due to sex difference in the developing brain [102], possibly related to biological differences in neurochemistry and hormone activity [103]. In addition, data from animal studies had shown that Mn exposure caused sex-dependent neuronal morphological change, and this change was not due to differential Mn accumulation between sexes but due to differences in sensitivity to Mn exposure [104]. All these differences may contribute to sex dimorphism in the associations between Mn exposure and neurodevelopment.

Our study incorporated the following limitations that warrant discussion. Firstly, most studies in this review are cross-sectional studies, so that no causal relationship can be inferred. In addition, a consistent biomarker for infants was not identified, perhaps teeth is the most promising biosample in this case, while being less easy to obtain than hair. Limitations in our data precluded us from identifying the safe range of manganese exposure and the periods of critical vulnerability of environmental manganese exposures. Finally, limited number of studies could be analyzed due to the relative homogeneity, however, we do not believe that this affected our analysis, given the stability of our sensitivity analysis.

Overall, to the best of our knowledge, this is the only comprehensive systemic review and meta-analysis regarding the biomarkers and sources of manganese exposure and cognitive, behavioral and motor functions in children. Outcomes from cohort studies and cross-sectional studies indicated that higher manganese exposures were negatively associated with neurodevelopment in children. In addition, this is the first meta-analysis for correlation between different manganese indicators where our results indicated that H-Mn was more significantly correlated with W-Mn than B-Mn. Therefore, we propose that hair is the most suitable biomarker in future studies.

## Conclusions

Higher manganese exposure is negatively associated with childhood neurodevelopment, especially cognitive and motor skills for children under 6 years old and cognitive and behavioral performance for children aged 6–18 years old. In the older group (6–18 years old), hair is the most reliable indicator of manganese exposure. However, evidence demonstrated sex difference upon manganese exposure while a clear pattern is not elucidated. Population based biomonitoring studies with standard cleaning methodologies of hair are warranted in order to set reference ranges of manganese in hair at different ages. Large prospective cohort studies are certainly warranted in order to support these results and identify the underlying biological mechanisms.

## Supplementary information

**Additional file 1.** PRISMA 2009 Checklist

**Additional file 2.** Evaluation of methodological quality of articles by using checklist in the Strengthening the Reporting of Observational Studies in Epidemiology Statement

**Additional file 3.** Characteristics of the articles included in the meta-analysis

**Additional file 4.** Sensitivity analysis was performed to evaluate the stability of the result

**Additional file 5.** Meta-analysis of studies reporting the effect of a 10-fold increase in drinking water manganese on intellectual quotient (IQ)

**Additional file 6.** Meta-analysis of studies reporting the effect of a e-fold increase in blood manganese on intellectual quotient (IQ)

**Additional file 7.** Correlations between manganese in biomarkers and environmental sample

**Additional file 8.** Meta-analysis of studies that stratified by sex reporting the effect of a 10-fold increase in hair manganese on intellectual quotient (IQ)

**Additional file 9.** The reference range or cut-off point used in the reviewed articles

## Abbreviations

AARES: The academic achievement records of the elementary schools
ADS: The attention-deficit/hyperactivity disorder (ADHD) diagnostic system
AMP: Aptitudes mentales primarias
APS: Accusway plus system
BASC-2: Behavior assessment system for children, 2nd edition
BOT-2: The bruininks-oseretsky test, 2nd edition
BSID: Bayley scales of infant and toddler development
CANTAB: Cambridge neuropsychological test automated battery
CAVLT: The children’s auditory verbal learning test
CBCL: The standardized child behavior checklist
CDIIT: The comprehensive developmental inventory for infants and toddlers
CPRS-R: The revised conners’ rating scale for parents
CPT-II: Conners’ continuous performance test ii version
CTRS-R: The revised conners’ rating scale for teachers
DBD: The disruptive behavior disorders
DDST-II: Denver developmental screening test II
DPD: Danish products developments
DS: Digit span
FT: Finger tapping
FTT: The forbidden toy task
GDI: Gesell developmental inventory
GP: Grooved pegboard
LNMB: Luria nebraska motor battery
MSCA: The McCarthy scales of children’s abilities
NBNA: Neonatal behavioral neurological assessments
NEPSY-II: Developmental neuropsychological assessment, second edition
NEUPSILIN-Inf: The Brazilian child brief neuropsychological assessment battery
PA: Pursuit aiming
PCM: The Raven’s progressive color matrices scale
PEDS: Parents’ evaluation of developmental status
RCPM: The Raven’s Colored progressive matrices
ROCF: The rey-osterrieth complex fig.
SA: Santa ana test
SDQ: The strengths and difficulties questionnaire
VRAM: The virtual radial arm maze
WASI: Wechsler abbreviated scale of intelligence
WISC: The wechsler intelligence scale for children
W-M: Woodcock-Muñoz tests of cognitive abilities
WRAVMA: The wide range assessment of visual motor abilities
