# Supplementary material for: Biomarkers of environmental manganese exposure and associations with childhood neurodevelopment: a systematic review and meta-analysis
Source: Environ Health. 2020 Oct 2;19:104. doi: 10.1186/s12940-020-00659-x (PMC7531154; doi:10.1186/s12940-020-00659-x)
Supplement: Supplementary file 2 — Additional file 2. Evaluation of methodological quality of articles by using checklist in the Strengthening the Reporting of Observational Studies in Epidemiology Statement [file 12940_2020_659_MOESM2_ESM.docx]

**Additional file 2.** Evaluation of methodological quality of articles by using checklist in the Strengthening the Reporting of Observational Studies in Epidemiology Statement

| Author, Year | Study Design | Setting | Participants | Variables | Data Sources/ Measurement | Bias | Study Size | Quantitative Variables | Statistical Methods | Total Items | Study Quality |
| --- | --- | --- | --- | --- | --- | --- | --- | --- | --- | --- | --- |
| Al-Saleh 2019 [41] | 1 | 1 | 1 | 1 | 1 | 1 | 1 | 1 | 0 | 8 | High |
| Bauer 2017 [64] | 0 | 1 | 1 | 1 | 1 | 1 | 1 | 1 | 0 | 7 | High |
| Betancourt 2015 [42] ‎ | 0 | 0 | 1 | 1 | 1 | 1 | 1 | 1 | 0 | 6 | Medium |
| Bhang 2013‎ [59] | 1 | 0 | 1 | 1 | 1 | 1 | 1 | 1 | 0 | 7 | High |
| Bouchard 2007 ‎[51] | 0 | 1 | 1 | 1 | 1 | 1 | 1 | 1 | 0 | 7 | High |
| Bouchard 2011‎ [16] | 1 | 1 | 1 | 1 | 1 | 1 | 1 | 1 | 0 | 8 | High |
| Bouchard 2018 [8] | 0 | 1 | 1 | 1 | 1 | 1 | 1 | 1 | 0 | 7 | High |
| Carvalho 2014 [22] | 1 | 0 | 1 | 1 | 1 | 1 | 1 | 1 | 0 | 7 | High |
| Carvalho 2018 [48]‎ | 1 | 1 | 1 | 1 | 1 | 1 | 1 | 1 | 0 | 8 | High |
| Chan 2015 [67] | 0 | 0 | 1 | 1 | 1 | 1 | 1 | 1 | 0 | 6 | Medium |
| Chiu 2017‎ [68] | 0 | 1 | 1 | 1 | 1 | 0 | 1 | 1 | 0 | 6 | Medium |
| Chung 2015 [36] | 1 | 1 | 1 | 1 | 1 | 1 | 1 | 1 | 1 | 9 | High |
| Claus Henn 2010‎ [35] | 1 | 1 | 1 | 1 | 1 | 1 | 1 | 1 | 1 | 9 | High |
| Claus Henn 2017 ‎[30] | 1 | 1 | 1 | 1 | 1 | 1 | 1 | 1 | 1 | 9 | High |
| Claus Henn 2018‎ [39] | 1 | 1 | 1 | 1 | 1 | 1 | 1 | 1 | 1 | 9 | High |
| Dion 2018‎ [20] | 1 | 1 | 1 | 1 | 1 | 1 | 1 | 1 | 0 | 8 | High |
| do Nascimento 2015 [44] | 0 | 0 | 1 | 1 | 1 | 1 | 1 | 1 | 0 | 6 | Medium |
| Ericson 2007 ‎[65] | 0 | 1 | 1 | 1 | 1 | 1 | 1 | 1 | 0 | 7 | High |
| Freire 2018 [32] | 1 | 1 | 1 | 1 | 1 | 1 | 1 | 1 | 0 | 8 | High |
| Frndak 2019 ‎[53] | 1 | 0 | 1 | 1 | 1 | 1 | 1 | 1 | 0 | 7 | High |
| Gunier 2015‎ [34] | 1 | 1 | 1 | 1 | 1 | 1 | 1 | 1 | 1 | 9 | High |
| Haynes 2015 ‎[45] | 1 | 1 | 1 | 1 | 1 | 1 | 0 | 1 | 0 | 7 | High |
| Haynes 2018 [7] | 0 | 1 | 1 | 1 | 1 | 1 | 0 | 1 | 0 | 6 | Medium |
| Hernandez-Bonilla 2011‎ [56] | 1 | 1 | 1 | 1 | 1 | 1 | 1 | 1 | 0 | 8 | High |
| Hernandez-Bonilla 2016 ‎[49] | 1 | 1 | 1 | 1 | 1 | 1 | 1 | 1 | 0 | 8 | High |
| Horton 2018‎ [66] | 1 | 1 | 1 | 1 | 1 | 1 | 1 | 1 | 0 | 8 | High |
| Khan 2011‎ [60] | 1 | 1 | 1 | 1 | 1 | 1 | 1 | 1 | 0 | 8 | High |
| Khan 2012 [71] | 1 | 0 | 1 | 1 | 1 | 1 | 1 | 1 | 0 | 7 | High |
| Kicinski 2015 ‎[61] | 0 | 1 | 1 | 1 | 1 | 1 | 1 | 1 | 0 | 7 | High |
| Kim 2009 ‎[62] | 1 | 1 | 1 | 1 | 1 | 1 | 1 | 1 | 0 | 8 | High |
| Lin 2013‎ [31] | 1 | 1 | 1 | 1 | 1 | 1 | 1 | 1 | 0 | 8 | High |
| Lucchini 2012a [54] | 1 | 0 | 1 | 1 | 1 | 1 | 1 | 1 | 0 | 7 | High |
| Lucchini 2012b [57] | 1 | 0 | 1 | 1 | 1 | 1 | 1 | 1 | 0 | 7 | High |
| Lucchini 2019 ‎[47] | 1 | 0 | 1 | 1 | 1 | 1 | 1 | 1 | 0 | 7 | High |
| Menezes-Filho 2011‎ [21] | 1 | 1 | 1 | 1 | 1 | 1 | 1 | 1 | 0 | 8 | High |
| Menezes-Filho 2014 ‎[52] | 1 | 0 | 1 | 1 | 1 | 1 | 1 | 1 | 0 | 7 | High |
| Mora 2015‎ [38] | 1 | 1 | 1 | 1 | 1 | 1 | 1 | 1 | 1 | 9 | High |
| Mora 2018 ‎[29] | 1 | 1 | 1 | 1 | 1 | 1 | 1 | 1 | 1 | 9 | High |
| Nascimento 2016 [46] | 0 | 0 | 1 | 1 | 1 | 1 | 1 | 1 | 0 | 6 | Medium |
| Oulhote 2014 [15] | 1 | 1 | 1 | 1 | 0 | 1 | 0 | 1 | 0 | 6 | Medium |
| Parvez 2011 ‎[63] | 1 | 1 | 1 | 1 | 1 | 1 | 1 | 1 | 0 | 8 | High |
| Rahman 2017 [69] | 1 | 1 | 1 | 1 | 1 | 1 | 1 | 1 | 0 | 8 | High |
| Rink 2014 ‎[40] | 0 | 0 | 1 | 1 | 1 | 1 | 1 | 1 | 0 | 6 | Medium |
| Riojas-Rodríguez ‎2010‎ [6] | 1 | 1 | 1 | 1 | 1 | 1 | 1 | 1 | 0 | 8 | High |
| Rodrigues 2016 [70] | 1 | 1 | 1 | 1 | 1 | 1 | 1 | 1 | 0 | 8 | High |
| Rugless 2014 ‎[55] | 0 | 1 | 1 | 1 | 1 | 1 | 1 | 1 | 0 | 7 | High |
| Takser 2003 [33]‎ | 1 | 0 | 0 | 1 | 0 | 0 | 1 | 1 | 0 | 4 | Medium |
| Torrente 2005 [58] | 0 | 0 | 1 | 0 | 0 | 0 | 0 | 1 | 0 | 2 | Low |
| Torres-Agustin ‎2013 [50]‎ | 1 | 1 | 1 | 1 | 1 | 1 | 1 | 1 | 0 | 8 | High |
| Wasserman 2006 ‎[9] | 1 | 1 | 1 | 1 | 1 | 1 | 1 | 1 | 0 | 8 | High |
| Wasserman 2016‎ [19] | 1 | 1 | 1 | 1 | 1 | 1 | 1 | 1 | 0 | 8 | High |
| Wright 2006 ‎[43] | 1 | 1 | 0 | 1 | 0 | 1 | 1 | 1 | 0 | 6 | Medium |
| Yu 2014 ‎[27] | 1 | 1 | 1 | 1 | 1 | 1 | 1 | 1 | 0 | 8 | High |
| Yu 2016 ‎[28] | 1 | 1 | 1 | 1 | 1 | 1 | 1 | 1 | 0 | 8 | High |
| Zhou 2019 ‎[37] | 1 | 1 | 1 | 1 | 1 | 1 | 1 | 1 | 0 | 8 | High |
