# Supplementary material for: Biomarkers of environmental manganese exposure and associations with childhood neurodevelopment: a systematic review and meta-analysis
Source: Environ Health. 2020 Oct 2;19:104. doi: 10.1186/s12940-020-00659-x (PMC7531154; doi:10.1186/s12940-020-00659-x)
Supplement: Supplementary file 3 — Additional file 3. Characteristics of the articles included in the meta-analysis [file 12940_2020_659_MOESM3_ESM.docx]

**Additional file 3.** Characteristics of the articles included in the meta-analysis

| Author, Year | Sources | Manganese Concentrations | Neurological Assessments | Transformation of Manganese Levels | *β* and 95% Confidence Interval or Standard Error | *n* | Adjustment for Covariates |
| --- | --- | --- | --- | --- | --- | --- | --- |
| Measured manganese in hair | | | | | | | |
| Bouchard 2011 [16] | Ground water | Hair: 0.7 (0.1-21)μg/g^a^ | WASI | Log_10_-transformed | Full Scale IQ: –3.3 (–6.1, –0.5), Verbal IQ: –2.7 (–5.4, 0.1), Performance IQ: –2.8 (–5.9, 0.4) | 302 | Maternal education and IQ, income, home stimulation score, and family structure, sex and age of child, IQ testing session, source of water and Fe concentration in tap water. |
| Bouchard 2018 [8] | Ground water | Hair: 0.3μg/g^b^ | WISC-IV | Log_10_-transformed | Full Scale IQ: –0.27 (–2.55, 2.02), Verbal IQ: 0.99 (–1.28, 3.26), Performance IQ: –1.01 (–3.43, 1.34) | 258 | Child’s age, maternal IQ, maternal education, income and IQ tester |
| Carvalho 2014 [22] | Air emissions from Fe-Mn alloy plant | Hair: 14.6 ± 11.8μg/g^c^ | WISC-III | Log_10_-transformed | IQ (estimated): –9.67 (–16.97, –2.37), Vocabulary: –0.74 (–2.53, 1.05), Digit Span: –2.59 (–4.13, –1.05), Block Design: –2.50 (–3.91,–1.10) | 70 | Maternal education |
| Dion 2018 [20] | Ground water | Hair: boy: 0.7μg/g^b^, girl: 0.7 μg/g^b^ | WASI | Log_2_- transformed | Full Scale IQ: boy: 1.3 (–0.3, 3.0), girl: –0.8 (–1.9, 0.3), Verbal IQ: boy: 0.9 (–1.1, 3.0), girl: –0.5 (–1.9, 0.9), Performance IQ: boy: 1.7 (–0.3, 3.7), girl: –1.1 (–2.4, 0.1) | Boy: 136, girl: 151 | Maternal IQ, maternal education and income |
| Menezes-Filho 2011 [21] | Fe-Mn alloy plant | Hair: 5.83 ± 11.5μg/g ^b^ | WISC-III | Log_10_-transformed | Full Scale IQ: –5.78 (–10.71, –0.21), Verbal IQ: –6.72 (–11.81, –0.63), Performance IQ: –2.41 (–7.39, 2.75) | 83 | Maternal education and nutritional status |
| Riojas-Rodríguez 2010 [6] | Air-borne Mn from mining district | Hair: control: 0.57μg/g^b^, exposed: 12.13μg/g^b^ | WISC-R | None | Full Scale IQ: –1.6 (–3.09, –0.1), Verbal IQ: –1.48 (–2.99, 0.04), Performance IQ: –1.45 (–3.16, 0.27) | 172 | Age, sex, Hb, Pb and maternal education |
| Wright 2006 [68] | Mining waste | Hair: 0.47 ± 0.46µg/g^c^ | WASI | None | Full Scale IQ: –0.01 (0.005), Verbal IQ: –0.12 (0.005), Performance IQ: –0.004 (0.006) | 31 | Sex and maternal education |
| Measured manganese in drinking water | | | | | | | |
| Bouchard 2011 [16] | Ground water | Ground water: 98μg/L^c^ | WASI | Log_10_-transformed | Full Scale IQ: –2.4 (–3.9, –0.9), Verbal IQ: –1.2 (–2.7, 0.3), Performance IQ: –3.1 (–4.9, –1.3) | 362 | Maternal education and IQ, income, home stimulation score, family structure, sex and age of child, IQ testing session, source of water and Fe concentration in tap water |
| Bouchard 2018 [8] | Ground water | Ground water: 62.1μg/L^c^ | WISC-IV | Log_10_-transformed | Full Scale IQ: 0.37 (–1.16,1.91), Verbal IQ: –0.25 (–1.65,1.14), Performance IQ: 0.87 (–0.69, 2.43) | 259 | Child’s age, maternal IQ, maternal education, income and IQ tester |
| Dion 2018 [20] | Ground water | Ground water: boy: 14.9μg/L^b^, girl: 14.2μg/L^b^ | WASI | Log_10_-transformed | Full Scale IQ: boy: 2.4 (0.3, 4.6), girl: –2.3 (–4.1, 0.6), Verbal IQ: boy: 1.0 (–1.7, 3.7), girl: –1.9 (–4.2, 0.3), Performance IQ: boy: 3.9 (1.4, 6.4), girl: –2.8 (–4.8, –0.8) | Boy: 136, girl: 151 | Maternal IQ, maternal education and income |
| Measured manganese in blood | | | | | | | |
| Haynes 2018 [7] | Air-borne Mn from Industry | Blood: 10.06 ± 1.30μg/L^b^ | WISC-IV | Log_e_-transformed | Full Scale IQ: 3.49 (–6.65,13.63), Working Memory: 7.12 (–4.14,18.37), Verbal Comprehension: –3.37 (–14.09, 7.36), Perceptual Reasoning: 3.46 (–5.88,12.80), Processing Speed: 2.08 (–5.72, 9.90) | 106 | Parent IQ |
| Wasserman 2016 [19] | Deep well water with reduced Mn | Blood: 14.8 ± 3.7μg/L^c^ | WISC-IV | Log_e_-transformed | Full Scale IQ: –3.27 (–7.51, 0.97), Working Memory: –6.27 (–11.94, –0.59), Verbal Comprehension: –3.37 (–14.09, 7.36), Perceptual Reasoning: –3.55 (–8.11, 1.02), Processing Speed: –0.18 (–4.86, 4.50) | 299 | Maternal IQ and maternal age, HOME score, child’s school grade, head circumference and plasma ferritin |

a: Median; b: Geometric mean; c:Mean ± standard deviation. Fe: Iron; Fe-Mn: Ferro-manganese; Hb: Hemoglobin; Mn: Manganese; Pb: Lead. HOME score: Home observation for measurement of the environment score; IQ: Intelligence Quotient.
