# Supplementary material for: Biomarkers of environmental manganese exposure and associations with childhood neurodevelopment: a systematic review and meta-analysis
Source: Environ Health. 2020 Oct 2;19:104. doi: 10.1186/s12940-020-00659-x (PMC7531154; doi:10.1186/s12940-020-00659-x)
Supplement: Supplementary file 4 — Additional file 4. Sensitivity analysis was performed to evaluate the stability of the result [file 12940_2020_659_MOESM4_ESM.docx]

**Additional file 4.** Sensitivity analysis was performed to evaluate the stability of the result

| Author, Year^#^ | Full Scale IQ | *I^2^* | *p* | Performance IQ‎ | *I^2^* | *p* | Verbal IQ | *I^2^* | *p* |
| --- | --- | --- | --- | --- | --- | --- | --- | --- | --- |
| None study is omitted | –2.51 ( –4.58, –0.45) | 59.8% | 0.015 | –1.79 (–3.11, –0.48) | 30.8% | 0.182 | –5.04 (–12.12, 2.04) | 97.9% | 0.000 |
| Bouchard 2011 [16] | –2.46 ( –4.92, 0.00) | 63.3% | 0.012 | –1.63 (–3.16, –0.10) | 38.6% | 0.135 | –5.39 (–13.58, 2.80) | 98.2% | 0.000 |
| Bouchard 2018 [8] | –3.10 ( –5.54, –0.66) | 59.0% | 0.023 | –1.95 (–3.55, –0.36) | 36.8% | 0.148 | –5.95 (–13.88, 1.99) | 97.9% | 0.000 |
| Carvalho 2014 [22] | –2.00 (–3.92, –0.08) | 53.9% | 0.043 | –1.52 (–3.22, 0.19) | 32.0% | 0.184 | –5.40 (–14.33, 3.53) | 97.9% | 0.000 |
| Dion 2018 (boy) [20] | –2.97 (–4.86, –1.08) | 50.5% | 0.059 | –2.05 (–3.00, –1.10) | 0.0% | 0.536 | –6.12 (–13.69, 1.45) | 98.2% | 0.000 |
| Dion 2018 (girl) [20] | –2.58 (–4.98, –0.17) | 65.2% | 0.008 | –1.61 (–3.04, –0.18) | 36.1% | 0.153 | –5.53 (–13.29, 2.24) | 98.2% | 0.000 |
| Menezes-Filho 2011 [21] | –2.16 (–4.31, –0.01) | 60.7% | 0.018 | –1.74 (–3.21, –0.28) | 40.4% | 0.122 | –4.80 (–12.54, 2.93) | 98.2% | 0.000 |
| Riojas-Rodríguez 2010 [6] | –2.17 (–4.16, –0.17) | 58.6% | 0.025 | –1.71 (–2.96, –0.45) | 28.3% | 0.212 | –4.52 (–12.08, 3.04) | 98.2% | 0.000 |
| Wright 2006 [43] | –2.92 (–5.69, –0.15) | 65.4% | 0.008 | –2.06 (–3.60, –0.52) | 31.0% | 0.192 | –1.98 (–4.07, 0.10) | 58.6% | 0.004 |

^#^: Given named study is omitted.
