# Supplementary figures and images for: Biomarkers of environmental manganese exposure and associations with childhood neurodevelopment: a systematic review and meta-analysis
Source: Environ Health. 2020 Oct 2;19:104. doi: 10.1186/s12940-020-00659-x (PMC7531154; doi:10.1186/s12940-020-00659-x)

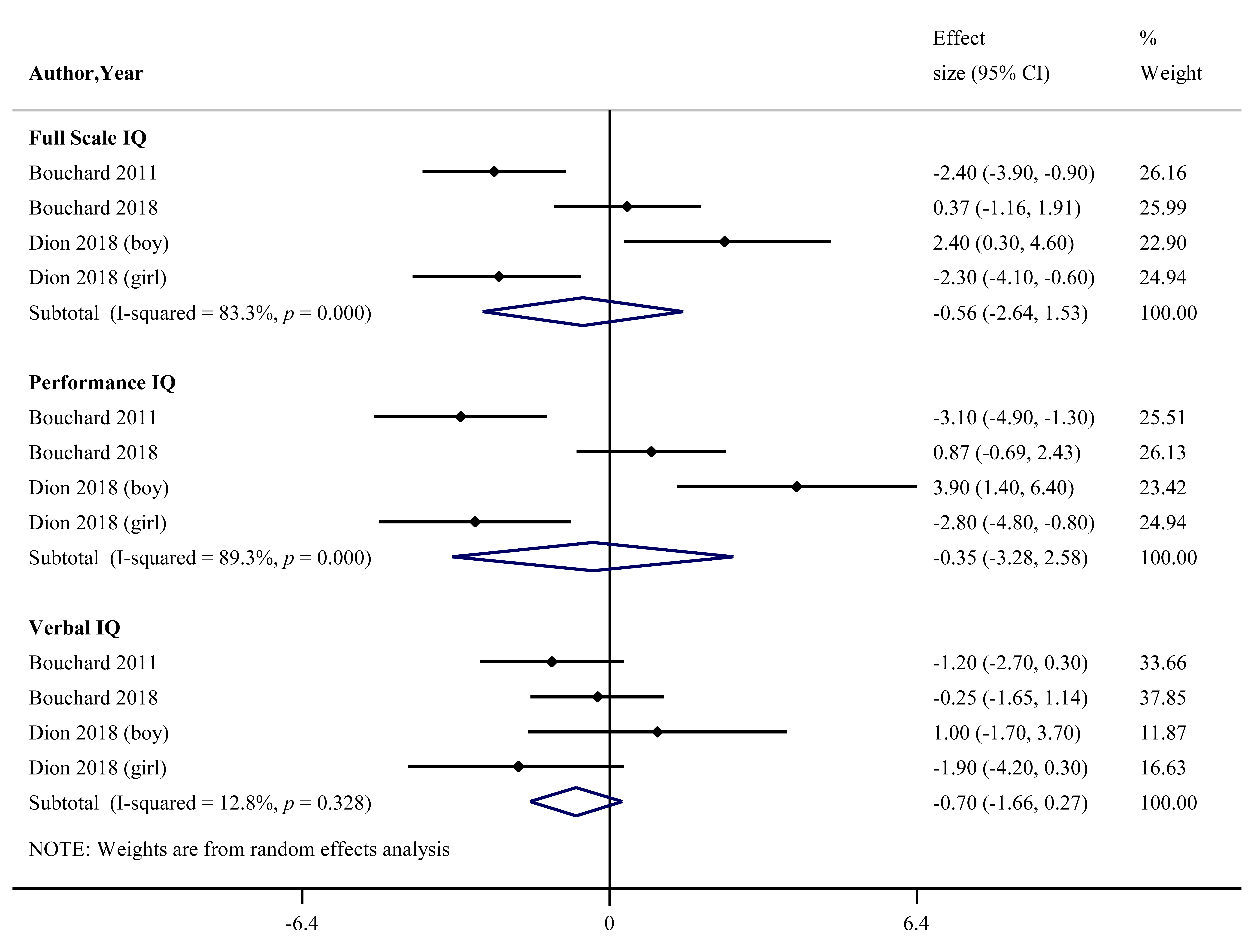

Supplement: Supplementary file 5 — Additional file 5. Meta-analysis of studies reporting the effect of a 10-fold increase in drinking water manganese on intellectual quotient (IQ) [file 12940_2020_659_MOESM5_ESM.tif]

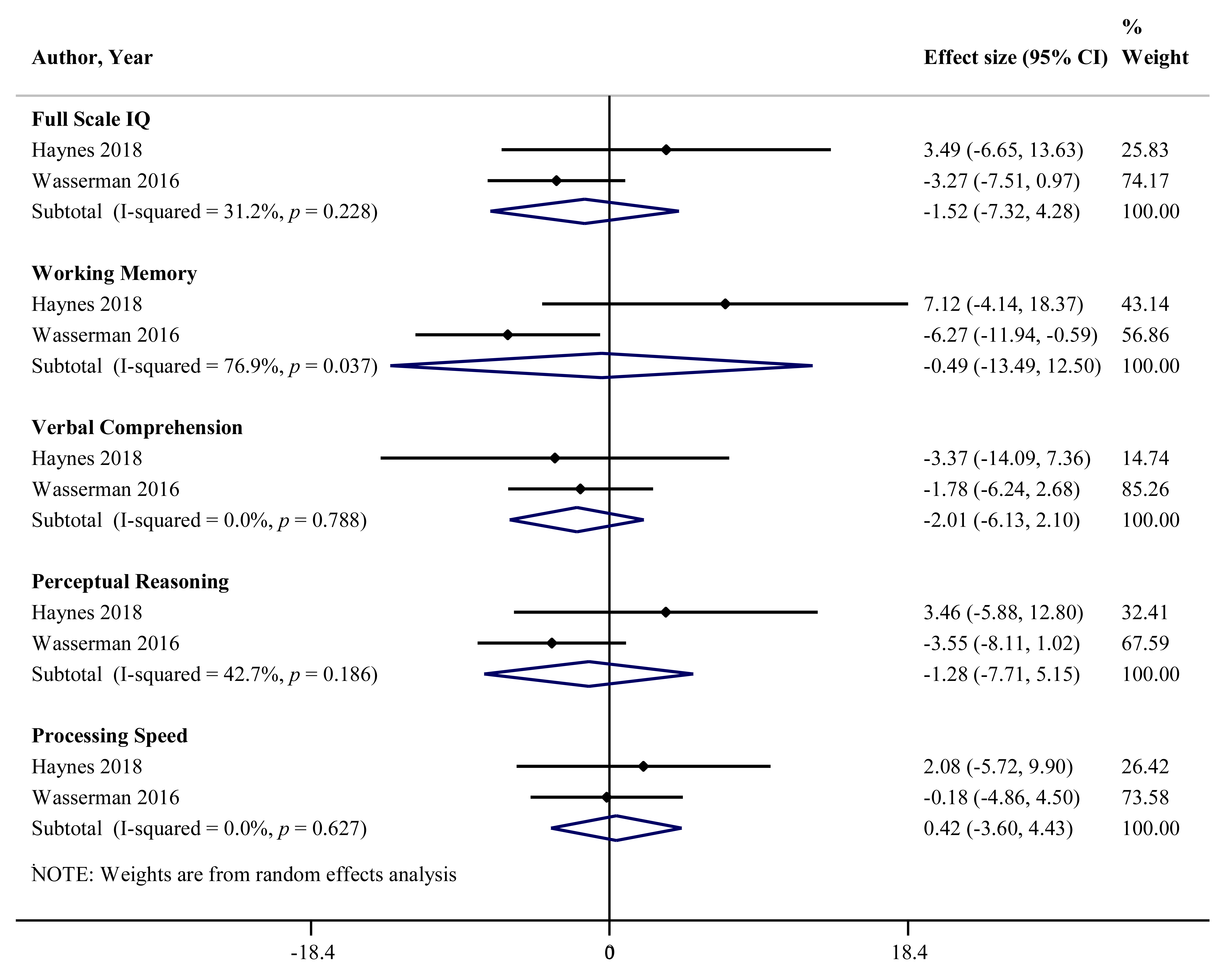

Supplement: Supplementary file 6 — Additional file 6. Meta-analysis of studies reporting the effect of a e-fold increase in blood manganese on intellectual quotient (IQ) [file 12940_2020_659_MOESM6_ESM.tif]

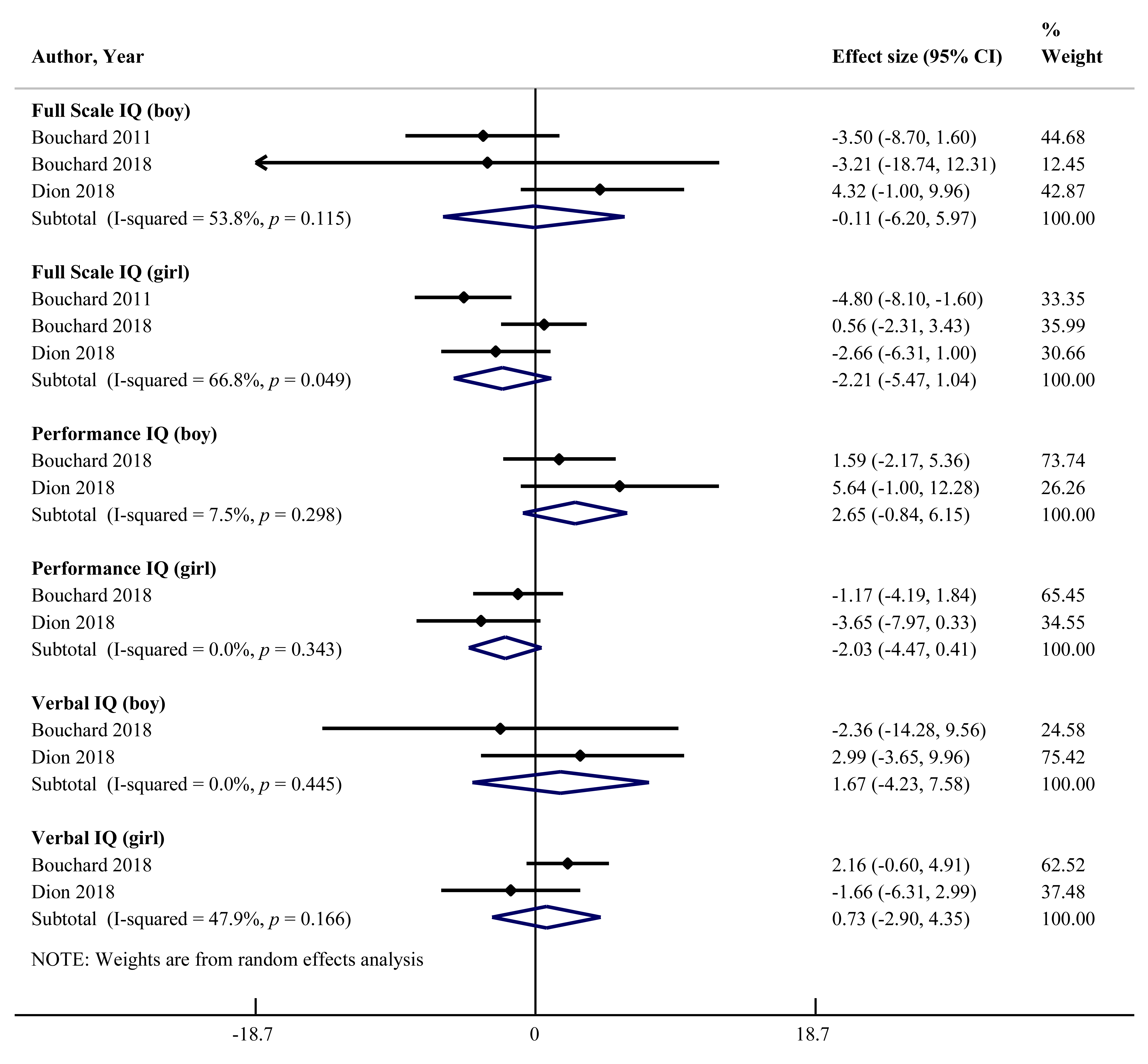

Supplement: Supplementary file 8 — Additional file 8. Meta-analysis of studies that stratified by sex reporting the effect of a 10-fold increase in hair manganese on intellectual quotient (IQ) [file 12940_2020_659_MOESM8_ESM.tif]
