# Supplementary material for: Biomarkers of environmental manganese exposure and associations with childhood neurodevelopment: a systematic review and meta-analysis
Source: Environ Health. 2020 Oct 2;19:104. doi: 10.1186/s12940-020-00659-x (PMC7531154; doi:10.1186/s12940-020-00659-x)
Supplement: Supplementary file 7 — Additional file 7. Correlations between manganese in biomarkers and environmental sample [file 12940_2020_659_MOESM7_ESM.docx]

**Additional file 7.** Correlations between manganese in biomarkers and environmental sample

| Author, Year | *r* | *n* | *p* | Fisher’s *z* | Standard Error |
| --- | --- | --- | --- | --- | --- |
| Correlations between manganese in hair and drinking water | | | | | |
| Dion 2018 [20] | 0.48 | 274 | <0.001 | 0.522984278 | 0.060745674 |
| do Nascimento 2015 [44] | 0.508 | 47 | 0.0003 | 0.560030416 | 0.150755672 |
| Nascimento 2016 [46] | 0.434 | 63 | <0.01 | 0.464814471 | 0.129099445 |
| Subtotal (95% CI) | 0.48 (0.40, 0.55) | - | - | 0.52 (0.42, 0.62) | - |
| Correlations between manganese in blood and drinking water | | | | | |
| Khan 2011 [60] | 0.05 | 201 | 0.56 | 0.050041729 | 0.071066905 |
| Nascimento 2016 [46] | 0.343 | 63 | <0.01 | 0.357488589 | 0.129099445 |
| Wasserman 2006 [9] | −0.04 | 95 | - | −0.040021354 | 0.104257207 |
| Subtotal (95% CI) | 0.11 (−0.09, 0.29) | - | - | 0.11 (−0.09, 0.30) | - |
| Correlations between manganese in hair and blood | | | | | |
| Haynes 2015 [45] | 0.002 | 370 | 0.97 | 0.002000003 | 0.052199575 |
| Haynes 2018 [7] | −0.27 | 98 | 0.03 | −0.276863823 | 0.102597835 |
| Menezes-Filho 2011 [21] | 0.053 | 70 | 0.66 | 0.053049709 | 0.122169444 |
| Nascimento 2016 [46] | 0.504 | 63 | <0.01 | 0.554653789 | 0.129099445 |
| Torres-Agustin 2013 [50] | 0.22 | 174 | <0.01 | 0.223656109 | 0.076471911 |
| Subtotal (95% CI) | 0.10 (−0.12, 0.31) | - | - | 0.10 (−0.12, 0.32) | - |
