# Supplementary material for: Biomarkers of environmental manganese exposure and associations with childhood neurodevelopment: a systematic review and meta-analysis
Source: Environ Health. 2020 Oct 2;19:104. doi: 10.1186/s12940-020-00659-x (PMC7531154; doi:10.1186/s12940-020-00659-x)
Supplement: Supplementary file 9 — Additional file 9. The reference range or cut-off point used in the reviewed articles [file 12940_2020_659_MOESM9_ESM.docx]

**Additional file 9.** The reference range or cut-off point used in the reviewed articles

| Author, Year | Biomarker/Source | Reference Value | Percentile | Number (Girls/Boys) | Age (Years) |
| --- | --- | --- | --- | --- | --- |
| Betancourt 2015 [42] | Hair | 2μg/g | - | 93 (46/47) | 11 |
| Bhang 2013 [59] | Blood | 8.154-21.453μg/L | 5-95th | 1001 (474/527) | 8-11 |
| Bouchard 2007 [51] | Hair | 3μg/g | - | 46 (22/24) | 6-15 |
| Chung 2015 [36] | Maternal blood | 20.0-30.0μg/L | - | 232 (124/108) | 6-month-olds |
| Claus Henn 2010 [35] | Blood | 20.2-28.0μg/L | 20-80th | 270 (131/139) | 12-month-olds |
| Haynes 2015 [45] | Hair | 0.21-0.75μg/g | 25-75th | 404 (187/217) | 7-9 |
| Haynes 2015 [45] | Blood | 8.2-11.2μg/L | 25-75th | 404 (187/217) | 7-9 |
| Khan 2012 [71] | Drinking water | 400μg/L | - | 201 (100/101) | 8-11 |
| Lin 2013 [31] | Cord blood | 59.34μg/L | 75th | 230 (102/128) | 2 |
| Rahman 2017 [69] | Drinking water | 200μg/L | - | 1265 (609/656) | 10 |
| Rodrigues 2016 [70] | Drinking water | 400μg/L | - | 525 (264/261) | 20-40 month-olds |
| Yu 2014 [27] | Cord serum | 5μg/L | - | 933 (439/494) | Newborns |
